# Virus and dsRNA-triggered transcriptional responses reveal key components of honey bee antiviral defense

**DOI:** 10.1038/s41598-017-06623-z

**Published:** 2017-07-25

**Authors:** Laura M. Brutscher, Katie F. Daughenbaugh, Michelle L. Flenniken

**Affiliations:** 1 Department of Plant Sciences and Plant Pathology, Montana State University, Bozeman, MT USA; 2 Department of Microbiology and Immunology, Montana State University, Bozeman, MT USA; 3 Pollinator Health Center, Montana State University, Bozeman, MT USA

## Abstract

Recent high annual losses of honey bee colonies are associated with many factors, including RNA virus infections. Honey bee antiviral responses include RNA interference and immune pathway activation, but their relative roles in antiviral defense are not well understood. To better characterize the mechanism(s) of honey bee antiviral defense, bees were infected with a model virus in the presence or absence of dsRNA, a virus associated molecular pattern. Regardless of sequence specificity, dsRNA reduced virus abundance. We utilized next generation sequencing to examine transcriptional responses triggered by virus and dsRNA at three time-points post-infection. Hundreds of genes exhibited differential expression in response to co-treatment of dsRNA and virus. Virus-infected bees had greater expression of genes involved in RNAi, Toll, Imd, and JAK-STAT pathways, but the majority of differentially expressed genes are not well characterized. To confirm the virus limiting role of two genes, including the well-characterized gene, *dicer*, and a probable uncharacterized *cyclin dependent kinase* in honey bees, we utilized RNAi to reduce their expression *in vivo* and determined that virus abundance increased, supporting their involvement in antiviral defense. Together, these results further our understanding of honey bee antiviral defense, particularly the role of a non-sequence specific dsRNA-mediated antiviral pathway.

## Introduction

Globally, honey bees (*Apis mellifera*) and other insects are important pollinators of plants in both natural and agricultural landscapes. Insect pollination services are valued worldwide at $175 billion annually^1^, and in the United States honey bee pollination is valued at $14.6 billion annually^2^. Commercially managed honey bee colonies, which are the primary pollinators of numerous agricultural crops, have experienced high annual mortality in the U.S. (i.e., 33% average annual loss since 2006) and parts of Europe^3–5^. Multiple abiotic and biotic factors, including pathogens, contribute to colony losses^6, 7^. Pathogen incidence and abundance have been positively correlated with Colony Collapse Disorder (CCD)-affected colonies in the U.S.^3, 6, 8^ and colony losses in different regions of North America, South America, and Europe^7, 9–17^. Honey bees are eusocial insects that live in colonies comprising approximately 40,000 sterile female workers, hundreds of male bees, and a single reproductive queen bee. Honey bees are often infected with pathogens including viruses, fungi, bacteria, and trypanosomatids, and they are typically parasitized by the *Varroa destructor* mite (reviewed in ref. 18).

The largest group of honey bee infecting pathogens are positive sense single-stranded viruses, including several Dicistroviruses (e.g., Israeli acute paralysis virus, Kashmir bee virus, Acute bee paralysis virus, and Black queen cell virus), Iflaviruses (e.g., Deformed wing virus, Sacbrood virus, and Slow bee paralysis virus), as well as taxonomically unclassified viruses (e.g., Chronic bee paralysis virus and the Lake Sinai virus group (reviewed in refs 19 and 20)). Honey bee-associated viruses exhibit variable pathogenicity and may cause deformity, paralysis, death, or remain asymptomatic (reviewed in refs 19 and 20). However, asymptomatic infections are commonly reported at levels of over 10^7^ virus equivalents (i.e., genomes and transcripts) per bee^21^, thus they likely affect bee physiology and health.

Like other insects, honey bee antiviral responses include autophagy, apoptosis, eicosanoid biosynthesis, endocytosis, melanization, the JAK/STAT (Janus Kinase/Signal Transducer and Activator of Transcription), Toll, NF-κB (Nuclear Factor κB), JNK (c-Jun N-terminal kinase), and MAPK (Mitogen-Activated Protein Kinases) pathways, and RNA interference (RNAi)^9, 15, 22–26^. RNAi is a post-transcriptional, sequence-specific, gene silencing mechanism and the small interfering RNA (siRNA)-mediated pathway is one of the primary insect antiviral defense mechanisms^27–37^. Correspondingly, several studies have shown that administration of virus-specific dsRNA or siRNA reduced viral load in honey bees^36, 38–40^. Furthermore, CCD-affected colonies had higher amounts of virus-specific 22 nt siRNAs as compared to non-CCD-affected colonies^37^ and early field studies suggested that honey bees fed IAPV-specific dsRNA had increased honey production and larger colony size^41^.

While experimental introduction of virus-specific dsRNA reduced honey bee virus infections, likely via RNAi^36, 38–40^, non-sequence-specific dsRNA (ns-dsRNA) has also been shown to reduce virus abundance and affect gene expression in honey bees and bumble bees^22, 42–45^. The more prominent role of ns-dsRNA mediated reduction in virus abundance in eusocial hymenopteran insects (e.g., honey bees and bumble bees), as compared to solitary insects (e.g., fruit flies and mosquitos) that do not exhibit this response, may reflect an evolutionary adaptation to limit virus transmission within colonies using general non-virus specific antiviral responses. In mammals, dsRNA serves as a virus-associated molecular pattern (VAMP) that is recognized by pathogen recognition receptors (PRRs), such as Toll-like receptor 3 (TLR3), Protein kinase R (PKR), Retinoic acid-inducible gene 1 (RIG-I), and Melanoma differentiation-associated gene 5 (MDA-5), and results in induction of the antiviral interferon response^46^. Similarly, Dicer, which is the endoribonuclease involved in RNAi, also serves as a dsRNA sensor that induces expression of antiviral defense genes (e.g., *vago*) in fruit flies, mosquitoes, and bumble bees^44, 47–49^. However, the role of specific genes in honey bee antiviral defense, particularly nonspecific dsRNA-mediated antiviral responses, are not well characterized.

Previously, we determined that treating honey bees with either virus sequence-specific-dsRNA (sp-dsRNA), or non-sequence specific dsRNA (ns-dsRNA), decreased virus abundance at 72 hours post-infection (hpi)^22^. To further investigate the mechanisms of dsRNA triggered antiviral defense and the dynamics of virus infection and corresponding immune responses in honey bees, we performed a time series experiment (i.e., 6, 48, and 72 hpi) that included transcriptional profiling of individual virus-infected and dsRNA-treated bees. We determined that honey bee gene expression varied with the progression of virus infection and included genes involved in endocytosis, development, transcriptional regulation, RNAi, and the Toll, Imd, JAK-STAT, and JNK pathways. Interestingly, bees that exhibited decreased virus abundance in the context of dsRNA treatment exhibited increased expression of two RNA helicases, one JNK pathway member, and several genes involved in dsRNA transport. Furthermore, we performed *in vivo* studies that confirmed the importance of the genes *dicer* and *cyclin*-*dependent serine/threonine kinase* (MF116383), which exhibited increased expression in virus-infected bees, in honey bee antiviral defense. Together these results further our understanding of honey bee antiviral defense mechanisms and the effects of dsRNA on honey bee gene expression and may lead to the development of strategies that limit virus infection in honey bees. Development and increased use of siRNAs and dsRNAs to reduce pathogens and pests (i.e., fungi, nematodes, and insects) in crops that are frequently visited by pollinators^50, 51^ also underscores the need to further examine the effects of these molecules on bee health.

## Results and Discussion

**Figure 1:**
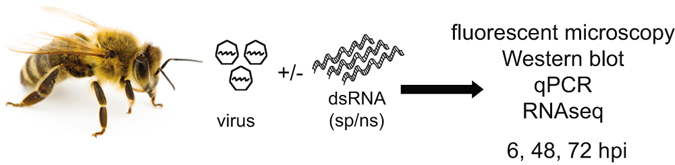
Schematic of honey bee virus infection trials. Schematic representation of the experiments performed to investigate honey bee antiviral immune mechanisms, including dsRNA triggered immune responses. Honey bees were infected with virus, SINV-GFP, and/or treated with dsRNA, which was composed of either virus-specific dsRNA (sp-dsRNA) or nonspecific dsRNA (ns-dsRNA). Bees were collected at 6, 48, and 72 hours post infection (hpi); virus abundance in individual bees was assessed by fluorescence microscopy, Western blot analysis, and qPCR. Honey bee gene expression was assessed using RNASeq. Honey bee image courtesy of Kathy Keatley Garvey; used with permission.

**Figure 2:**
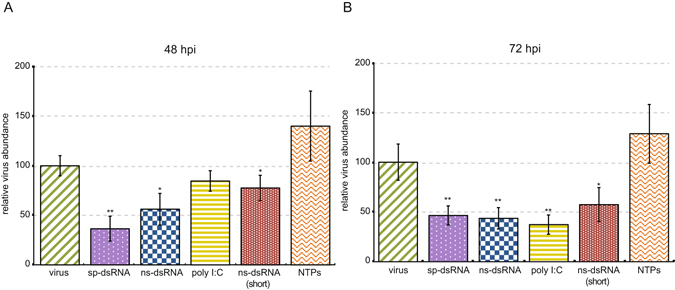
Relative virus RNA abundance was reduced in dsRNA-treated bees as compared to virus-infected bees. Relative abundance of SINV-GFP in individual bees (n = 10) was assessed by qPCR. (**A**) At 48 hours post-infection (hpi), bees treated with dsRNA (1 kb) had reduced relative virus RNA abundance (includes both virus genomes and transcripts) by 64% for sp-dsRNA-treated bees (dotted purple, **p < 0.005) and 44% for ns-dsRNA-treated bees (checkered blue, *p < 0.05), as compared to bees infected with virus only (green stripes). Likewise, bees treated with short dsRNA (0.5 kb) had 24% less virus (dotted red, *p < 0.05) than virus-infected bees. (**B**) At 72 hpi bees treated with dsRNA had reduced relative virus abundance by 54% (**p < 0.005) and 56% (**p < 0.005) for sp-dsRNA and ns-dsRNA, respectively. Similarly, bees treated with poly(I:C) (yellow stripes), a structural analog of dsRNA, had 63% less virus than virus-infected bees (**p < 0.005). Bees treated with short dsRNA also had 43% less virus (dotted red, *p < 0.05) than bees infected with virus alone. The virus abundance in bees treated with NTPs (orange wavy lines) was not significantly different from virus-infected bees at either 48 hpi (A) or 72 hpi (**B**). Percent relative virus abundance for each sample was determined via ΔΔCT analysis (using *Am rpl8* as the house keeping gene); statistical differences between treatment and virus-infected bees were performed using one-sided Student’s t-tests, *p ≤ 0.05, **p ≤ 0.005. The bars represent the standard error of the mean.

Honey bees are commonly infected by positive sense single-stranded RNA (+ssRNA) viruses, which replicate via a dsRNA intermediate. RNAi mediated by virus-specific dsRNA is an important antiviral defense mechanism in honey bees^9, 22, 36, 38–41^, but nonspecific dsRNA-triggered antiviral response pathway(s) also play a role in honey bee antiviral defense^22^. To further investigate honey bee antiviral defense mechanisms, including RNAi and nonspecific dsRNA-mediated mechanisms^22^, we infected bees with a model virus in the presence or absence of dsRNA (Fig. 1). There are currently no honey bee virus isolates or infectious clones, so we utilized a recombinant Sindbis virus that expresses GFP (SINV-GFP) as a tractable model of virus infection in honey bees^22^. SINV-GFP inoculations were performed via intrathoracic injection in the absence or presence of multiple species and lengths of dsRNA, including virus-specific dsRNA (sp-dsRNA, 928 bp), nonspecific dsRNA corresponding to Drosophila C virus sequence (ns-dsRNA, 1,017 bp), and luciferase sequence (dsRNA-short, 355 bp). In addition, co-administration of polyinosinic-polycytidylic acid (poly(I:C)), a synthetic mimic of dsRNA, or nucleoside triphosphates (NTPs), served as positive and negative controls, respectively; mock-infected controls were also performed. Bees from each experimental treatment group were collected at 6, 48, and 72 hpi, a time course that allowed for assessment of both early and late antiviral responses. Relative virus abundance was examined via fluorescence microscopy and quantified based on relative protein and RNA abundance using Western blot analyses and quantitative PCR (qPCR), respectively (Figs 1 and 2 and S1 and S2).

### Virus abundance reduced in dsRNA-treated honey bees

Honey bees treated with sp-dsRNA or ns-dsRNA had reduced virus abundance. Fluorescent microscopy of bees that were infected with SINV-GFP in the absence or presence of dsRNA provided qualitative evidence that dsRNA treatment reduced SINV-GFP at 72 hpi (Supplementary Fig. S1). To more quantitatively examine the reduction of SINV-GFP, we performed Western blot analyses of individual bee lysates at 72 hpi, which determined that dsRNA, regardless of sequence composition, reduced virus abundance at the protein level (Supplementary Fig. S2). Virus abundance was most accurately measured and compared by assessing relative RNA abundance via qPCR (Supplementary Table S1 and Supplementary Fig. S3). At 48 hpi and 72 hpi, bees treated with sp-dsRNA, ns-dsRNA, and poly(I:C) had decreased relative SINV-GFP abundance as compared to bees infected with virus only and bees simultaneously treated with virus and NTPs (Figs 2 and S4). At 48 hpi, the relative virus abundances of sp-dsRNA and ns-dsRNA treated bees were reduced by 64% (p < 0.005) and 44% (p < 0.05), respectively, as compared to bees infected with virus only (Fig. 2A). At 72 hpi, the relative virus abundances of sp-dsRNA- and ns-dsRNA-treated bees were reduced by 54% (p < 0.005) and 56% (p < 0.005) as compared to bees infected with virus only (Fig. 2B). Bees treated with poly(I:C) at 72 hpi had reduced virus abundance by 63% (p < 0.005). Reduced relative virus abundance in dsRNA-treated bees was also observed in additional biological replicates, which included virus-infected bees at 48 and 72 hpi from two additional honey bee colonies for a total of n = 30 per treatment (Supplementary Fig. S4).

### Transcriptional level evaluation of virus and dsRNA induced immune responses in honey bees

The transcriptional profiles of virus-infected honey bees are indicative of the cellular pathways and mechanisms that are regulated in response to virus infection. Likewise, we hypothesized that a subset of the differentially expressed genes would also be regulated in response to dsRNA, a VAMP. To further elucidate the honey bee transcriptional response to virus infection and the mechanisms of dsRNA triggered antiviral defense, we performed transcriptome profiling (RNASeq) of individual virus-infected bees, bees infected with virus in the presence of sp-dsRNA or ns-dsRNA, dsRNA-treated bees in the absence of virus, and mock-infected bees at 6, 48, and 72 hpi (Fig. 1). Forty-seven individual bee RNASeq libraries were prepared using the Illumina TruSeq Stranded RNA Sample Prep kit and paired-end sequenced (2 × 100 nt) on an Illumina HiSeq. 2500, resulting in an average of 12 million reads per individual bee sample (Supplementary Table S2). On average, 77% of reads mapped to the *A*. *mellifera* genome assembly 4.5 from NCBI^52^. Prior to sequencing, bees were screened for confounding pre-existing infections via pathogen-specific PCR and qPCR in order to identify individuals with little to no preexisting infections (Supplementary Tables S1 and S3).

### Genes differentially expressed in virus-infected bees and dsRNA-treated bees

**Figure 3:**
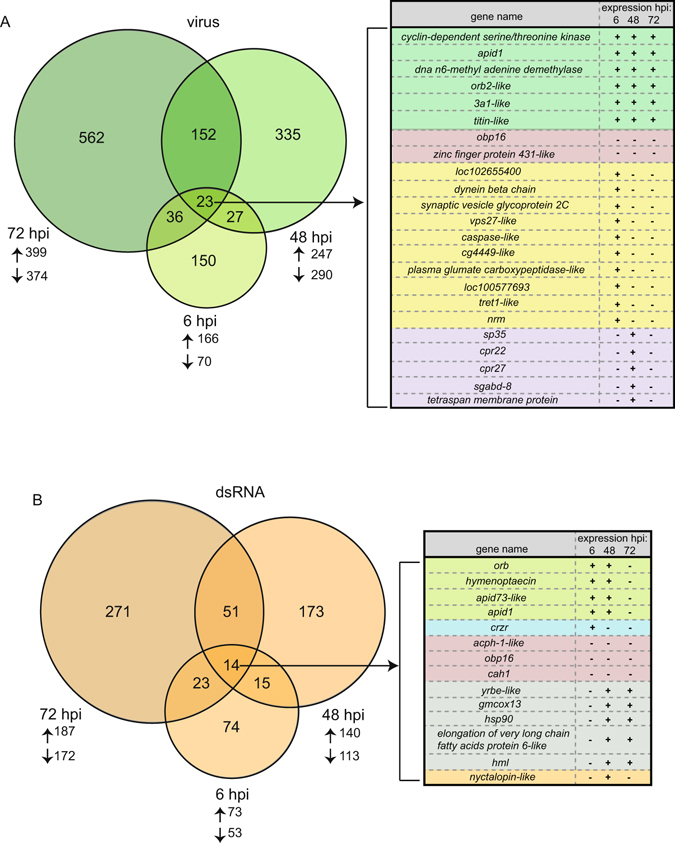
Honey bee transcriptional response to virus-infection and dsRNA-treatment is time-dependent. (**A**) There were hundreds of differentially expressed genes (DEGs) in virus-infected bees, as compared to mock-infected bees. Venn diagram analysis identified shared and unique DEGs of virus-infected bees 6, 48, and 72 hours post-infection (hpi). Twenty-three genes were differentially expressed at all three time points post-infection. Six of these genes consistently exhibited increased expression (highlighted in green and listed from highest average log_2_ fold change to lowest) and two genes consistently exhibited decreased expression (red). Ten genes exhibited increased expression at 6 hpi and decreased expression at 48 and 72 hpi (yellow). Five genes (purple) displayed increased expression at 48 hpi, but decreased expression at 6 and 72 hpi. Arrows pointing up denote number of genes that exhibited increased expression for each time point and arrows pointing down denote number of genes that exhibited decreased expression. (**B**) There were hundreds of differentially expressed genes (DEGs) in dsRNA-treated bees compared to mock-infected bees. Venn diagram analysis identified shared and unique DEGs of dsRNA-treated bees at 6, 48, and 72 hpi, including 14 genes that were differentially expressed at all time points. There were four genes (green) that exhibited increased expression at 6 and 48 hpi and decreased expression at 72 hpi, three of which are antimicrobial peptides (AMPs). One gene, *crzr*, exhibited increased expression at 6 hpi, but decreased expression in bees 48 and 72 hpi (blue). Three genes displayed consistently decreased expression (red). Five genes had decreased expression at 6 hpi and increased expression at 48 and 72 hpi (gray), including *heat shock protein 90*. One gene, *nyctalopin*-*like*, exhibited decreased expression in bees 6 and 72 hpi, but increased expression in bees 48 hpi (orange). DEGs with Benjamini-Hochberg corrected q-values ≤ 0.05 were included in Venn diagram analyses. Full lists of DEGs and their fold changes from all contrasts in each Venn diagram are provided in Supplementary Tables S7 and S8.

Transcriptome analysis of virus-infected bees over the course of infection (i.e., 6, 48, and 72 hpi) determined that virus-infection altered the expression of hundreds of genes as compared to mock-infected bees (Figs 3A and S5). The majority of differentially expressed genes (DEGs) in virus-infected and dsRNA-treated bees are not well characterized or do not have known roles in antiviral defense (Supplementary Table S5). Genes that exhibited increased expression at 6 hpi were functionally enriched for the biological processes phosphorylation and transcriptional regulation (Supplementary Fig. S6). The genes with increased expression at 48 hpi were enriched in transcriptional regulation, cell adhesion, immune responses, and cellular migration (Supplementary Fig. S6 and Supplementary Table S5). Similarly, virus-infected bees 72 hpi also exhibited increased expression of genes enriched for transcriptional regulation and gene silencing (Supplementary Fig. S6). Genes involved in morphogenesis were differentially expressed throughout all time points (Supplementary Fig. S6)^53^.

Venn diagram analysis demonstrated that the honey bee transcriptional response to virus infection varies with time post-infection (Fig. 3A). As time post-infection increased, so did the number of DEGs, from 236 DEGs to 773 DEGs (Fig. 3A). Twenty-three genes were commonly differentially expressed throughout the course of the infection (Fig. 3A), though only eight of these genes were differentially expressed in a uniform direction (Fig. 3A and Supplementary Tables S4, S5 and S7). Six of these genes exhibited increased expression including an uncharacterized transcript encoding a probable *cyclin*-*dependent serine/threonine kinase* (MF116383), *apid1*, *DNA* *n6*-*methyl adenine demethylase* (*loc412878*), *orb2*-*like*, *solute carrier organic anion transporter family member 3a1*-*like* (*sloc3a1*), and *titin*-*like* (Fig. 3A and Supplementary Tables S4, S5 and S7). Two genes had lower expression in all virus-infected bees: *obp16* and *zinc finger protein 431*-*like*.

Many viruses generate long dsRNA molecules during their replication cycle. Long dsRNA molecules are not a typical product of eukaryotic gene expression, so they serve as triggers of eukaryotic antiviral immune responses (e.g., RNAi and interferon responses)^46, 54^. To further investigate the role of dsRNA stimulation in honey bee antiviral defense, we examined changes in gene expression over time. The genes that exhibited increased expression in dsRNA treated bees 48 hours after treatment were enriched for functions including oxidation-reduction, cellular morphogenesis, and immune response (Supplementary Table S5). Bees 72 hours post-treatment exhibited increased expression of genes enriched for cellular morphogenesis, transcriptional regulation, vesicle-mediated transport, and RNA interference (Supplementary Table S5), paralleling the results of a previous study that examined the effects of nonspecific dsRNA (GFP-dsRNA) on honey bee gene expression^43^.

Venn diagram analysis of dsRNA-treated bees 6, 48, and 72 hours post-treatment identified 14 shared DEGs, three of which exhibited decreased expression: *carbonic anhydrase 1*, *venom acid phosphatase acph*-*1*-*like*, and *odorant binding protein 16*, which also exhibited decreased expression in virus-infected bees (Fig. 3B, Supplementary Tables S5 and S8). Similar to virus-infected bees, *heat shock protein 90* (*hsp90*) was also differentially expressed throughout all dsRNA-treated bees. In dsRNA-treated bees 6 and 48 hpi, several genes encoding antimicrobial peptides (i.e., *apidaecin*, *apidaecins type 73*-*like*, *abaecin* and *hymenoptaecin*) exhibited increased expression (Fig. 3B), most of which also exhibited increased expression in virus-infected bees (Supplementary Table S6). In addition, both virus-infected and dsRNA-treated bees 48 and 72 hpi exhibited increased expression of *scavenger receptor class c* (*scr*-*c*), which plays a role in dsRNA uptake in *D*. *melanogaster* ^55^ and may play an analogous role in honey bees. Together, our analyses indicate that dsRNA-treatment alters gene expression in honey bees, and that there are common and unique aspects between differential gene expression in virus-infected bees and virus-infection in the context of dsRNA.

#### qPCR validation of RNASeq results

In order to validate RNAseq results, we examined the relative expression of fourteen genes (i.e., *cyclin-dependent kinase*, *orb2-like*, *titin-like*, *DNA n6-methyl adenine demethylase*, *slco31-like*, *hsp90*, *abaecin*, *ago2*, *dicer*, *igfn3-10*, *mfs-transporter*, *jra*, *fam102b*) that exhibited increased expression in virus and/or dsRNA treated bees at 48 and/or 72 hpi via qPCR of sequenced bees 72 hpi (Supplementary Fig. S7). The expression of ten of those genes was also examined by qPCR in sequenced bees at 48 hpi (Supplementary Fig. S7). All but two of the fourteen genes assayed (*igfn3-10* and *titin-like*) were confirmed to have increased expression in virus-infected and/or dsRNA-treated bees via qPCR (Supplementary Fig. S7). Several genes (e.g., *hsp90*, *cyclin-dependent kinase*, *ago2*, *dicer*, *mfs-transporter*, *formin-j*) were also confirmed to have increased expression in biological replicate experiments that utilized pooled virus-infected honey bee samples (72 hpi) from two different colonies, likely with different genetic backgrounds (Supplementary Fig. S8). Together these results confirm the RNASeq results and provid further evidence to their importance in honey bee antiviral defense.

### Differentially expressed genes in a cellular context

**Figure 4:**
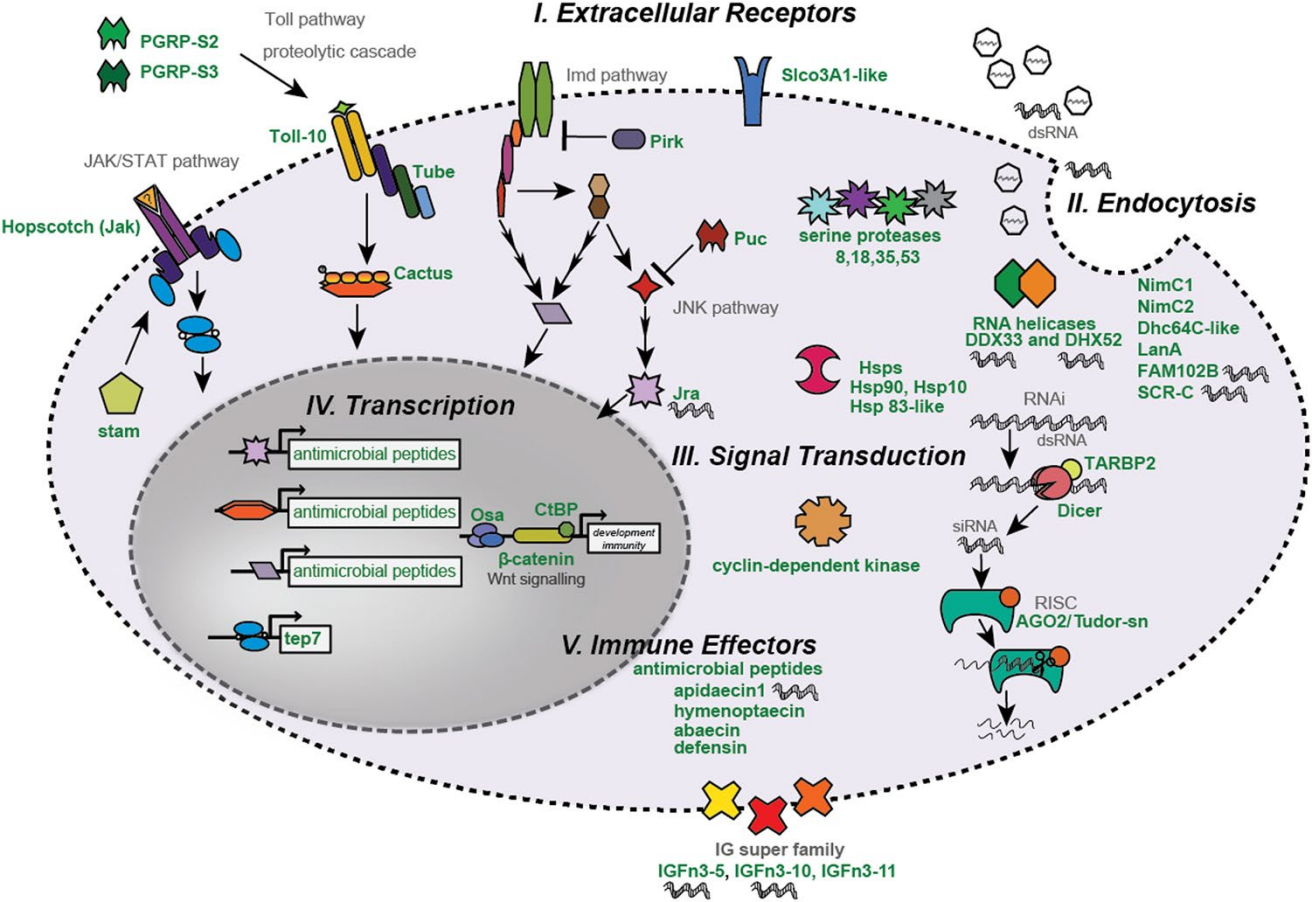
Differentially expressed genes in response to virus and/or dsRNA treatment in a cellular context. The DEGs of virus-infected and dsRNA-treated honey bees were surveyed for differential expression of genes involved in previously characterized insect immune pathways: RNAi, Toll, JAK/STAT (Janus Kinase and Signal Transducer and Activator of Transcription), Imd (Immune Deficiency), and JNK (c-Jun N-terminal kinases)^29, 56^. This analysis determined that many genes encoding extracellular receptors, proteins involved in endocytosis, signal transduction, and immune responses exhibited increased expression; these DEGs (denoted by bold and green font) are depicted in a cellular context. Many genes exhibited higher fold change in bees treated with both virus and dsRNA as compared to bees infected with virus only (denoted with dsRNA), suggesting their involvement in dsRNA-triggered immune responses. Complete DEG lists are provided in Supplementary Table S6.

To compare our results with what is currently known about insect immunity, we surveyed the DEGs of virus-infected and dsRNA-treated bees for genes involved in previously characterized insect immune pathways. This analysis determined that many genes encoding extracellular receptors and proteins involved in endocytosis, signal transduction, as well as immune effector proteins (e.g., antimicrobial peptides) exhibited increased expression (Fig. 4 and Supplementary Table S6). Some of the genes identified herein are illustrated in a cellular context in order to illustrate their potential functions in antiviral defense (Fig. 4).

#### Extracellular receptors and transporters

In the context of virus infection, extracellular receptors can serve in pathogen recognition and host defense or be co-opted by the virus to facilitate entry. The membrane localized *solute carrier organic anion transporter family member 3a1*-*like* (*sloc3a1*) consistently exhibited increased expression in virus-infected bees (i.e., 1.6–2.3 fold increase) (Figs 3A and 4 and Supplementary Tables S4, S5 and S7). Interestingly, *slco3a1* also exhibited increased expression in SBV and DWV infected bees^25^. The JAK/STAT receptor, *hopscotch*, exhibited increased expression in virus-infected bees (Fig. 4 and Supplementary Table S6). The JAK/STAT pathway is involved in both insect development and antiviral defense^29, 56^. Additionally, two pathogen recognition receptors of the Toll pathway, *peptidoglycan receptor s2* (*pgrp*-*s2*) and *peptidoglycan receptor s3* (*pgrp*-*s3*), and the toll-like receptor *toll*-*10* exhibited increased expression in virus-infected bees (Fig. 4 and Supplementary Table S6). Similar to virus-infected bees, bees treated with only dsRNA also exhibited greater expression of *toll*-*10* and *pgrp*-*s3*. The Toll pathway is primarily involved in defense against Gram-positive bacteria and fungi, but has also been implicated in antiviral defense in *D*. *melanogaster* and *Aedes aegypti* ^56^. Similar to the Toll pathway, activation of the Imd pathway results in antimicrobial peptide production, but it is typically activated by Peptidoglycan recognition protein LC (PGRP-LC) binding to the diaminopimelic-containing peptidoglycan of Gram-negative bacteria^29^. The Imd pathway is also important for fruit fly antiviral defense against some viruses, including Sindbis virus^56–58^.

Lastly, three genes encoding immunoglobulin domain containing proteins (i.e., *igfn3*-*5*, *igfn3*-*10*, and *igfn3*-*11*) exhibited increased expression in virus-infected bees (Fig. 4 and Supplementary Table S6). In insects, immunoglobulin domain containing proteins serve in a variety of functions, including cell to cell adhesion, pathogen recognition, and promotion of phagocytosis^59^. Similarly, hemolin, an immunoglobulin-domain containing protein exclusively expressed in lepidopterans, exhibits increased expression in Chinese Oak Silk moths treated with dsRNA or infected by Baculovirus^60^.

#### Endocytosis

Endocytosis, including phagocytosis, is an immune effector function carried out by hemocytes^61^ and may also be important for systemic RNAi^55, 62^. In addition, many viruses exploit endocytic pathways for entry^63^. SINV and other alphaviruses typically enter cells via receptor binding followed by clathrin-mediated endocytosis^64^. Several genes involved in phagocytosis, including *nimc1*, *nimc2*, *dhc64c*-*like*, and *laminin a*, exhibited increased expression in virus-infected bees (Fig. 4 and Supplementary Table S6). Laminins aid in cellular adhesion, migration, differentiation, and morphology^65^. In mammalian cells, SINV utilizes a laminin receptor for viral entry^65^. Likewise, virus-infected honey bees had greater expression of a JAK/STAT effector molecule *thioester protein 7* (*tep7*). In mosquitoes, thioester proteins bind to invading bacteria which promotes phagocytosis of these pathogens^66^, but thioesters are also associated with improved defense against Dengue and West Nile viruses via unknown mechanisms^67^.

In *D*. *melanogaster* S2 cells, genes involved in receptor-mediated endocytosis are important for dsRNA uptake, including the genes *scavenger receptor c* (*scr*-*c*), *fam102b*, and *sap*-*r* ^55^. Bees treated with virus, dsRNA, or both exhibited increased expression of *scr*-*c*, but bees treated with both virus and dsRNA exhibited the greatest increase (Fig. 4 and Supplementary Table S5). Additionally, the expression of *fam102b* was significantly increased in bees that were treated with both virus and dsRNA. In contrast, *sap*-*r*, which is a protease associated with late stage endosomes, exhibited decreased expression in bees treated with both virus and dsRNA and may be indicative of virus-specific dsRNA triggered modification of endosomal development^68^, although future investigation is required.

#### Signal transduction cascades

Signal transduction cascades are the means by which a chemical or physical signal is transmitted through a cell resulting in a response. For example, detection of pathogen associated molecular patterns (PAMPs), including dsRNA, results in activation of cellular transduction cascades that activate particular immune responses^69^. In our data set, reads aligning to *Apis mellifera* LOC25387, which encodes a previously uncharacterized transcript (MF116383) that has high sequence homology to an Eastern honey bee (*Apis cerana*) probable *cyclin*-*dependent serine/threonine kinase* (XM_017051141.1), exhibited the greatest increase in expression in virus-infected bees as compared to mock-infected controls (i.e., 5.7–13 fold increase) (Figs. 3A and 4, Supplementary Fig. S9, and Supplementary Tables S4, S5, and S7)^9^. In general, cyclin-dependent serine/threonine kinases are activated by cyclins and phosphorylate serine and threonine residues of substrate proteins, resulting in regulation of cell cycle progression and transcription. Though, the specific proteins that interact with this *cyclin*-*dependent serine/threonine kinase* are unknown. Likewise the expression of *Am* LOC25387 transcripts were increased in DWV and SBV co-infected bees and IAPV-infected bees^9, 25^.

Genes involved in Toll pathway signal transduction also exhibited increased expression in virus-infected bees including *cactus 1* and *cactus 2*, which suppress NF-κB signaling, and *tube*, an adaptor protein that promotes NF-κB signaling (Fig. 4 and Supplementary Table S5). In *Drosophila*, immune pathways are tightly regulated in order to balance immune responses, thus increased expression of pathway inhibitors (e.g., *cactus 2*) does not necessarily indicate complete or continuous repression of the pathway (e.g., Toll)^70^. Likewise, we determined that *pirk*, which represses Imd pathway signaling^71, 72^, exhibited increased expression in virus-infected bees (Fig. 4 and Supplementary Table S5). Intriguingly, JNK pathway activation is often linked with Imd pathway activation^56^. The transcriptional effector of the JNK pathway, *jun*-*related antigen* (*jra*), had greater expression in bees that were both virus-infected and treated with dsRNA at 6 and 48 hpi, and increased expression in all virus-infected groups at 72 hpi (Supplementary Fig. S7 and Supplementary Table S5). Bees treated with only dsRNA followed similar, but lower, expression patterns as compared to mock-infected bees, suggesting that the JNK signaling may be involved in dsRNA-triggered responses.

The Wnt/beta-catenin signaling pathway, which is involved in cellular proliferation and differentiation, has also been implicated in insect host-virus interactions, though its role in immune function is less well characterized^73, 74^. Several genes involved in Wnt signaling (e.g., *osa*) exhibited increased expression in virus-infected bees (Fig. 4 and Supplementary Table S5). The involvement of the Wnt signaling pathway in honey bee antiviral defense has also been implicated in the context of IAPV infection^9^, thus it is likely that Wnt signaling is important to honey bee antiviral defense.

#### Immune effector proteins

Several antimicrobial peptides, which are effector molecules of Toll, Imd, and JNK pathways, exhibited increased expression in virus-infected and dsRNA-treated bees. Importantly, *apidaecin 1* exhibited increased expression in all virus-infected bees and bees treated with dsRNA alone (Figs 3 and 4 and Supplementary Tables S4–S8)^56, 75^. Apidaecins are proline-rich antimicrobial peptides (AMPs) that have bactericidal activity against Gram-negative bacteria^75, 76^. Virus-infected and/or dsRNA-treated bees also exhibited increased expression of *abaecin* and *hymentoptaecin* (Supplementary Table S5), indicating activation the Imd and/or JNK pathways^56, 75, 77^. Increased AMP expression in virus-infected honey bees and other insects has previously been reported, though their role in antiviral defense is not yet understood^24–26, 78^. It may be that AMPs do not have a direct role in antiviral defense and that increased transcript levels of AMPs and genes involved in pathogen recognition and signal transduction (e.g., *pgrp*-*s3*) simply indicate activation of these pathways^56, 75, 79^. The activation of Toll, Imd, and JNK signal transduction cascades likely stimulate transcription of hundreds of genes, including antiviral effectors that await further characterization.

Heat shock proteins (Hsps) are involved in general stress responses and protein degradation and stabilization. In fruit flies, these ubiquitously expressed proteins are important for defense against some viruses^80, 81^. Our transcriptional level analysis identified several genes encoding heat shock and accessory proteins that exhibited increased expression in virus-infected bees including *hsp90*, *activator of hsp90*, *60 kda hsp*, *10 kda hsp*, *hsp83*-*like*, and *hsf5* (Supplementary Table S5). Hsp90 expression was also increased in dsRNA-treated bees. In *Drosophila*, Hsp90 binds to and stabilizes the RNA-induced silencing complex (RISC) as part of the RNAi response^82, 83^, but Hsp90 can also be exploited by both insect and human viruses (e.g., Flock House virus and Polio virus) in order to stabilize RNA replication^84, 85^. Future studies aimed at better understanding the functions of heat shock proteins, particularly Hsp90, in virus-infected honey bees will be exciting since these proteins may either be antagonistic or beneficial to specific viruses.

RNA interference is an important antiviral and post-transcriptional gene regulatory mechanism in honey bees that is initiated by Dicer recognition of dsRNA^22, 36, 37, 39^. Notably, there was greater expression of genes involved in RNAi (i.e., *argonaute*-*2* (*ago2*), *dicer*, *tudor*-*sn*, *hsc70*-*4*, and *tarbp2*) in virus-infected bees (Fig. 4 and Supplementary Table S6). Interestingly, enhanced expression of *dicer* and *ago*-*2* in virus-infected honey bees was observed in another study^24^, whereas increased expression of genes involved in RNAi has not been observed in virus-infected fruit flies^49^. In our studies, administration of dsRNA, in the absence of virus infection, did not induce *Apis mellifera dicer* or *ago2* expression, indicating that VAMP immune triggering does not completely recapitulate the immune response to virus infection. Additional studies are required to better understand the mechanisms of transcriptional activation of genes involved in honey bee RNAi^86^.

The role of DExD box RNA helicases in honey bee antiviral defense is particularly interesting because in mammals many of these proteins function as nonspecific cytosolic sensors of dsRNA (e.g., MDA-5 and RIG-I), which activate the antiviral interferon response^46, 49^. In *Culex pipiens f*. *molestus* mosquitoes, *D*. *melanogaster*, and *Bombus terrestris*, Dicer-2 serves as a dsRNA pathogen recognition receptor (PRR), that after binding dsRNA, results in the increased activation of antiviral immune effectors (e.g., *vago*)^29, 42, 44, 47, 49^. DWV-infected honey bees exhibited increased expression of *Apis mellifera vago* (*loc503505*), but differential *vago* expression was not observed in our data set; many host factors (e.g., age/life stage) may be involved, but perhaps *vago* expression is only increased in response to specific honey bee infecting viruses (Supplementary Table S5). The expression of two RNA helicases (i.e., *rna helicase ddx33 and rna helicase dhx52*) was increased in virus-infected and dsRNA-treated honey bees (Fig. 4 and Supplementary Table S5). RNA helicase DHX33 has been identified as a dsRNA receptor in mammals that when bound to dsRNA or bacterial RNA, activates NLRP3 inflammasome-mediated interferon stimulation^87^, but RNA Helicase DDX33 has not been implicated in dsRNA-immunostimulation in insects. Future exploration of the role of these important dsRNA sensors in activating antiviral response in honey bees will likely lead to the discovery of analogous pathways in other organisms.

### Reduced expression of *dicer* and *cyclin*-*dependent kinase* enhanced virus abundance *in vivo* and confirmed their role in limiting virus infection in honey bees

**Figure 5:**
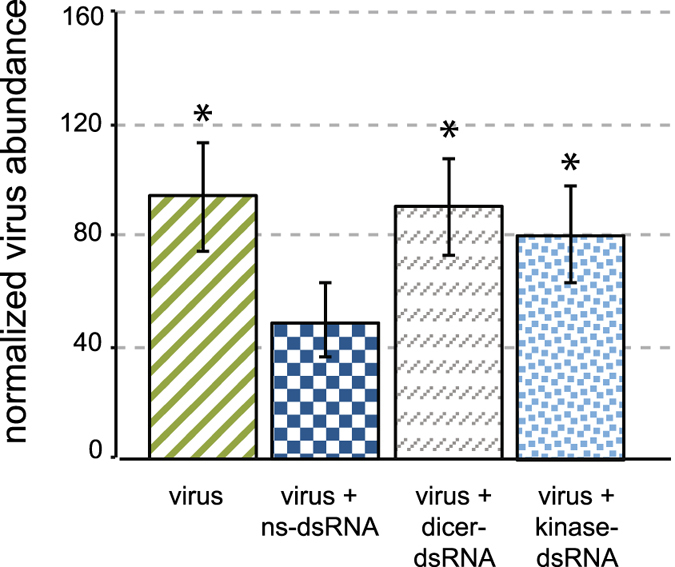
Reduced expression of two honey bee genes resulted in increased virus abundance. To further investigate the role of *dicer and cyclin*-*dependent kinase* in honey bee antiviral defense, we utilized RNAi-mediated gene knock down to reduce their expression (Supplementary Fig. S10) and qPCR to determine the impact on virus abundance. SINV-GFP abundance in *kinase* and *dicer* specific dsRNA-treated bees at 72 hpi was increased by 48% (*p < 0.05) and 44% (*p < 0.05), respectively, compared to the ns-dsRNA control, which is the most relevant comparison given that administration of dsRNA, including the dsRNAs used to reduce the expression of dicer and cyclin-dependent kinase reduces virus abundance. Percent relative virus abundance for each sample was determined via qPCR and ΔΔCT analysis using *Am rpl8* as the house keeping gene. Statistical differences between the ns-dsRNA control group and the other treatments were determined using one-sided Student’s t-tests, *p ≤ 0.05, **p ≤ 0.005. The bars are standard error of the mean.

In order to further investigate the biological importance of two putative antiviral genes, *dicer* and a probable *cyclin*-*dependent serine/threonine kinase* (MF116383), we utilized RNAi-mediated gene knock down to reduce their expression and investigate the impact on virus abundance. We expected that reduced expression of these antiviral genes would result in increased virus abundance, as compared to the virus abundance in bees treated with ns-dsRNA. Virus-infected bees treated with *cyclin*-*dependent kinase*-specific dsRNA exhibited decreased expression by 48 hpi (40%) and 72 hpi (30%) compared to respective controls (i.e., virus-infected and ns-dsRNA treated bees) (Supplementary Fig. 10). At 72 hpi, virus abundance in bees with reduced *cyclin*-*dependent kinase* levels was higher compared to the virus abundance in bees treated with ns-dsRNA, 77% versus 43% relative virus abundance (p < 0.05) (Fig. 5). Bees 48 hpi followed similar trends (Supplementary Fig. 10). Bees treated with *dicer*-specific dsRNA in the context of virus infection exhibited reduced expression of *dicer* at 48 hpi, but not 72 hpi (Supplementary Fig. 10). Though the kinetics of *dicer* knock-down differed from the probable *cyclin*-*dependent kinase*, bees treated with *dicer* specfic dsRNA had a greater abundance of virus compared to bees treated with ns-dsRNA, 90% versus 43% (Fig. 5). These results confirm the role of *dicer* in limiting virus infections in honey bees and highlight the importance of a previously uncharacterized transcript encoding a probable kinase, *cyclin*-*dependent kinase* (MF116383), in limiting virus infection. Further investigation of this probable *cyclin*-*dependent kinase* and the proteins with which it interacts may lead to the discovery of novel honey bee antiviral pathways or aid in further characterization of known immune pathways.

### Synthesis of honey bee transcriptional response to virus infection

This is the first study to examine individual honey bee antiviral responses to infection with controlled inoculum of a model virus at multiple time points. Other studies have examined honey bee responses to virus infection at the transcriptional level, but they vary by virus-challenge methodologies (e.g., mite vectored, infection via injection, and oral infection), purity and strain of virus inoculum, tissues examined, post-infection assay time, and bee developmental stage, which reduce commonalities in transcriptional results between studies^9, 15, 22, 24–26, 88–91^.

**Figure 6:**
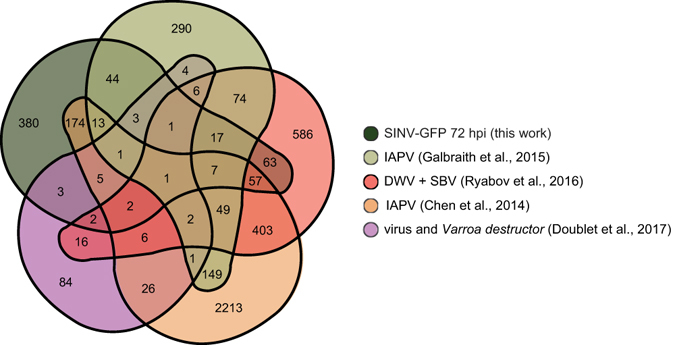
Venn diagram of shared and unique DEGs in bees infected with viruses from this and other studies. The DEGs identified in SINV-GFP-infected bees 72 hpi were compared to those identified in naturally IAPV-infected bees (Chen *et al*. 2014, orange)^9^, bees infected with IAPV via oral inoculation (Galbraith *et al*., 2015, green)^24^, SBV and DWV-infected bees (Ryabov *et al*., 2016, pink)^25^, and a common DEG list that was compiled from 19 gene expression data sets including *Varroa destructor*-parasitized and virus-infected bees (Doublet *et al*., 2017)^92^ (purple). Lists of DEGs from all studies that were used to generate the Venn diagram^131^ are provided in Supplementary Tables S14 and Venn diagram results are listed in S15.

In spite of the methodological differences between this and other honey bee transcriptional level analyses, we identified common DEGs associated with virus infected honey bees. Using Venn diagram analysis, we compared our DEG lists to DEGs of symptomatic IAPV-fed bees^24^, SBV and DWV-infected bees^25^, adult honey bees naturally infected with IAPV^9^, and a synthesis of common DEGs that was recently generated from 19 gene expression data sets from *Varroa destructor*-parasitized and virus-infected bees^92^ (Fig. 6, Supplementary Tables S14 and S15). There was one DEG that was shared in all five DEG lists: *protein lethal*(*2*)*essential for life*-*like*, which encodes a protein in the small heat shock protein (Hsp 20) family, further supporting the role of heat shock proteins in honey bee antiviral defense (Supplementary Table S15)^93^. Additional comparisons, between this study and other transcriptome dat﻿a, indicated that there were many shared DEGs involved in the Toll, Imd, JAK/STAT, JNK, and RNAi pathways, as well numerous uncharacterized pathways (Supplementary Tables S14 and S15)^9, 22, 24, 26, 94^. There were 87 shared DEGs between virus-infected bees at 72 hpi (this work) and symptomatic IAPV-fed bees (Fig. 6 and Supplementary Table S15), including increased expression of three genes involved in RNAi (i.e., *ago2*, *dicer*, and *tar rna*-*binding protein 2*)^24^. A few AMPs also exhibited differential expression in many of the DEG lists. For example, *hymenoptaecin* exhibited differential expression in SINV-GFP-infected bees, DWV and SBV co-infected bees, and in the virus and *Varroa destructor* DEG synthesis^25, 92^ (Fig. 6 and Supplementary Table S15). The gene encoding for Apidaecin exhibited differential expression in SINV-GFP-infected bees, symptomatic IAPV-fed bees, SBV and DWV-infected bees, and adult honey bees naturally infected with IAPV^9, 24, 25^. Notably, one of the top ranked genes with decreased expression in the transcriptome synthesis was *zinc finger protein 431*-*like* ^92^ (Fig. 6). Our study also determined that this gene had decreased expression in most virus-infected and dsRNA co-treated bees at all time points (Fig. 3). The members of the Pit-Oct-Unc (POU) family have a wide variety of functions primarily involved in the neuroendocrine system^95^. This may correspond with work showing that pathogen infections induce neuronal and behavioral changes (e.g., premature foraging behavior) in honey bees, which may function as a form of social immunity in insect societies^96^.

## Summary

Managed honey bee colonies in the US and parts of Europe have experienced high annual mortality levels (i.e, 33% average in US since 2006)^4, 5, 11^. In addition to other factors, several studies indicate that colony losses correlate with high pathogen incidence and abundance^7, 9–17^, including infection by (+)ssRNA viruses^20, 56^. The outcome of virus infection is influenced by many factors^97^, including virus strain^98^, agrochemical exposure^97^, nutritional status^99^, genetic diversity of the colony^88, 100–104^, colony management, mite parasitism^15, 89, 105^, co-infections^106, 107^, and immune responses at both the colony and individual levels. Honey bee antiviral responses include canonical immune pathways (e.g., Toll, JAK/STAT, Imd, JNK), RNAi, and nonspecific dsRNA-mediated immune pathways, though the relative roles of these pathways and the mechanistic details of honey bee antiviral immune responses are not thoroughly understood.

This is the first study to examine both individual and temporal honey bee transcriptional response to virus infection. Our results further indicate that honey bee antiviral defense includes canonical insect immune pathways, RNAi, and a nonspecific dsRNA-mediated antiviral defense mechanism. Transcriptional analysis of dsRNA-treated bees showed that dsRNA results in increased expression of genes involved in the JNK pathway, RNA helicases, and dsRNA transport, which parallels dsRNA transport and response mechanisms in fruit flies and mammals. While the results described herein implicate the role of numerous genes, several biological processes, and the involvement of Dicer and a probable cyclin-dependent kinase (MF116383), which had greatest sequence similarity with an *Apis cerana* gene (XM_017051141.1), in honey bee antiviral defense, future studies are required to further elucidate the honey bee antiviral defense network. Better understanding of honey bee antiviral defense mechanisms may aid in the development of strategies that reduce honey bee colony losses and furthers our knowledge of antiviral immune responses in insects, which may ultimately reveal evolutionary conserved pathways in other organisms.

## Methods

### Honey bees

Frames of newly emerging bees were obtained from honey bee colonies maintained at Montana State University in Bozeman, MT, USA. Young (~24 hours post-emergence) female worker bees were utilized for experiments. The bees were housed in modified deli-containers at 32 °C and were provided water and bee candy^22, 108^.

### Sindbis virus (SINV-GFP) infection trials

There are currently no infectious honey bee virus clones, but studies with semi-purified honey bee virus preparations have provided valuable information^24, 25, 56^. We utilized a recombinant model virus, Sindbis virus expressing green fluorescent protein (SINV-GFP)^22, 109^. There are several advantages to utilizing this virus including the ability to control the dose of virus inoculum, monitor the progression of virus infection using GFP, and the assurance that the honey bees were not previously infected with, nor exposed to, SINV-GFP. In addition, Sindbis virus does not encode a suppressor of RNAi (VSR)^110^. We and others have used SINV-GFP to investigate honey bee^22^, fruit fly^109^, and mosquito^111^ antiviral defense mechanisms, thus facilitating comparison of immune responses in both natural mosquito hosts and non-native hosts (i.e., honey bee and fruit fly) that have not co-evolved with this virus. Honey bees were immobilized via incubation at 4 °C for 20 minutes and injected in the thorax with 3,750 plaque forming units (PFUs) of SINV-GFP^22^ diluted in 2 μl of 10 mM Tris buffer (pH 7.5) using a Harbo large capacity syringe equipped with disposable needles (Honey Bee Insemination Service; http://www.honeybeeinsemination.com/equipment2.html). The needles were prepared from borosilicate capillary tubes (0.8–1.10 × 100 mm) with a micropipette puller (Narishige Model PC-10, East Meadow, New York, USA). To investigate the role of dsRNA in honey bee antiviral defense, SINV-GFP was inoculated with different types of dsRNA (1 μg each), including virus-specific dsRNA (sp-dsRNA, 928 bp), nonspecific dsRNA matching Drosophila C virus sequence (ns-dsRNA, 1,017 bp), or luciferase sequence (LUC dsRNA, 355 bp) (Supplementary Table S1). Bees were also co-injected with 1 μg high molecular weight polyinosinic-polycytidylic acid (poly(I:C)), InvivoGen) or 1 μg nucleoside triphosphates (NTP), the positive and negative controls. Mock-infection controls were also performed. Bees were collected at 6, 48, or 72 hours post-infection (hpi); a time frame that allowed for virus dissemination and infection, while maintaining optimal conditions for bees housed within the laboratory setting^22^.

### dsRNA preparation

dsRNA was generated by *in vitro* transcription with T7 RNA polymerase^109, 112^. T7 promoter containing dsDNA PCR-products were amplified using primers listed in Supplementary Table S1, with the following thermocycler program: pre-incubation of 95 °C (5 min), 35 cycles of 95 °C (30 s), 60 °C (30 s), and 72 °C (1 min) followed by a final incubation at 72 °C (5 min). PCR products were templates for T7 polymerase transcription (100 μl reactions: NTPs (each 7.5 mM final), RNase OUT (40 units) (Invitrogen), buffer (400 mM HEPES pH 7.5, 120 mM MgCl_2_, 10 mM Spermidine, 200 mM DTT); reactions were carried out at 37 °C overnight (8–10 hours). DNA was removed by incubating with RQ1 DNAse (1 unit; Promega) for 15 minutes at 37 °C. ssRNA products were ethanol precipitated, suspended in 200 μl Rnase-free water, and annealed at 100 °C for 5 minutes and then slowly cooled to room temperature. dsRNA products were purified by phenol:chloroform extraction and ethanol precipitation. Quality was assessed by agarose gel electrophoresis and spectrophotometry. The dsRNA quantity based on gel band intensity was assessed using ImageJ^113^.

### dsRNA-mediated gene knockdown

The expression of two candidate antiviral genes: *dicer* (XM_016917734.1) and a novel transcript with 91% sequence identity with the *A*. *cerana* probable cyclin-dependent serine/threonine-protein kinase DDB_G0292550 (XM_017051141.1) was reduced by RNAi-mediated gene knockdown. In order to assess the effects of gene knockdown on virus abundance, bees were infected with SINV-GFP (using methods as above) and co-injected with 1 μg of either gene-specific or nonspecific (DCV-specific) dsRNA (control) (Supplementary Table S1).

### Honey bee RNA isolation and purification

TRizol reagent (Invitrogen) was added to individual bee thorax and abdomen homogenate and RNA was isolated following the manufacturer’s instructions. Prior to gene expression analysis by RNASeq or qPCR, RNA was further purified using Qiagen RNAeasy columns including on-column DNase t reatment (Qiagen) to remove DNA from samples. RNA was quantified using a spectrophotometer.

### Reverse transcription/cDNA synthesis

Reverse transcription reactions (25 μl) were performed using 500 ng of total RNA and random hexamer primers (500 ng) (IDT, Coralville, IA) incubated with Maloney murine leukemia virus (M-MLV) reverse transcriptase (Promega, Madison, WI) for 1 hour at 37 °C, according to the manufacturer’s instructions.

### Quantitative PCR (qPCR)

Quantitative PCR was utilized to examine the relative abundance of virus and honey bee host gene expression in each sample using previously described methods that are in accordance with published guidelines^114^. All qPCR reactions were performed in triplicate using 2 μL of cDNA as template. Each 20 μl reaction was composed of cDNA template, 1X SYBR Green (Invitrogen), 1X Choice Taq Master Mix (Denville Scientific Inc.), 3 mM MgCl_2_, and forward and reverse primers (600 nM each). A CFX Connect Real Time instrument (BioRad) was utilized for qPCR, the thermo-profile for virus (e.g., SINV-GFP and BQCV) and *A*. *mellifera rpl8* analyses consisted of a single pre-incubation 95 °C (3 min), 40 cycles of 95 °C (5 s), 60 °C (20 s), 72 ºC (30 s), and a final elongation 72 ºC (4﻿ min)(Supplementary Table S1). Positive and negative controls, including the use of RNA templates from no RT enzyme cDNA reactions, were included for all qPCR analyses and exhibited the expected results.

To quantify the viral RNA (i.e., genome and transcript) abundance in each sample target SINV-GFP qPCR amplicons were cloned into the pGEM-T (Promega) vector, as described in Flenniken and Andino *et al*.^22^. Plasmid standards, containing 10^9^ to 10^3^ copies per reaction, were used as qPCR templates to assess primer efficiency and generate the standard curve used for viral genome copy quantification^22^. The qPCR primers for RNAseq validation were designed using Primer3Plus and with 60 °C annealing temperatures^115^ (Supplementary Table S1). Melt point analysis and 2% agarose gel electrophoresis ensured qPCR specificity^116^. Primer efficiencies were evaluated using qPCR assays of cDNA and plasmid dilution series, and calculated by plotting log_10_ of the concentration versus the crossing point threshold (C(t)) values and using the primer efficiency equation, (10^(1/Slope)−1)^ × 100) (Supplementary Table S16).

The ΔΔC(t) method was used to calculate relative abundance of SINV-GFP in individual bees (n = 10) because it was most accurate; the ΔΔC(t) method ensures that results are not skewed by inadvertent differences in RNA reverse-transcription efficiencies and starting cDNA template abundance^114, 116, 117^. The ΔC(t) for each sample was calculated by subtracting the *Am rpl8* C(t) from the SINV-GFP C(t). The honey bee gene encoding ribosomal protein 8, *Am rpl8*, was selected as an appropriate housekeeping gene for qPCR because it has been utilized in several other studies^118–121^ and analysis of the RNASeq data presented herein confirmed that *rpl8* expression levels were similar in all sequenced libraries. The ΔΔC(t) was calculated by subtracting the average virus-infected ΔC(t) values from the ΔCt values for each treatment group. For host gene expression analyses and RNAseq validation, the percent gene expression for each gene of interest (GOI) was calculated using the following formula: 2^−ΔΔC(t)^ × 100 = % gene expression, in which ΔC(t) = GOI C(t)- *rpl8* C(t), and ΔΔC(t) = sample ΔC(t) – mock-infected control ΔC(t). Based on previous work^22, 44^, we hypothesized that bees (n = 10) co-injected with dsRNA or poly(I:C) would have decreased relative virus abundance as compared to the virus-only treated group. To examine relative virus abundance between treatment groups (e.g., virus-infected bees and dsRNA or poly(I:C) co-treated bees) that had equal variance and normal distribution we performed one-tailed Student’s t-tests. Analysis of honey bee host gene expression revealed unequal variance between treatments groups and thus Welch’s t-tests were used to identify statistical differences in host gene expression.

### RNAseq Library Preparation and Sequencing

Individual bee cDNA was screened for pre-existing infections via PCR for several honey bee pathogens (Supplementary Table S3) using the PCR thermocycler protocol: 95 °C (5 min); 35 cycles of 95 °C (30 s), 57 °C (30 s), and 72 °C (30 s), followed by final elongation at 72 °C for 4 minutes. If the sample was positive for a pathogen, the quantity was then assessed using qPCR. The RNA isolated from the abdomens of at least three representative bees with low (<2,000 DWV and/or BQCV virus genome copies versus 7 × 10^4^–7 × 10^6^ SINV-GFP copies) to no pre-existing infections for each treatment group and time point were selected for transcriptome sequencing for a total of 47 individual bees (Supplementary Table S3).

Prior to RNASeq library preparation, RNA from each sample was further purified and DNase treated using Qiagen RNeasy columns. RNA quality was assessed using an Agilent 2200 Bioanalyzer and quantified via spectrophotometer. RNA was sent to the Roy J. Carver Biotechnology Center at the University of Illinois for library preparation (Illumina TruSeq Stranded RNA Sample Prep kit). Libraries were prepared and pooled by experimental time point and quantitated using an Illumina Library quantification kit (Kapa). Each pool was paired-end sequenced (2 × 100 nt) on a HiSeq. 2500 using a TruSeq SBS sequencing kit version 4, yielding ~12 million reads per sample, corresponding to at least 9.7 fold coverage (Supplementary Table S2), which is in the range of coverage reported in other honey bee transcriptome studies^24, 25, 122^. Sequence data was deposited into the NCBI Sequence Read Archive under accession number SRP101337 and is linked with NCBI BioProject #PRJNA377749.

FastQC and fastx-toolkit were used to remove low quality reads (<Q30). Illumina adapters were trimmed with Trimmomatic^123^, and reads were aligned to the *A*. *mellifera* genome assembly 4.5 from NCBI with Tophat v2.0.14^52^; on average, ~77% of reads from each sample were mapped (Supplementary Table S2). The normalized number of Fragments Per Kilobase of transcript per Million mapped reads (FPKM) was determined using CuffDiff^124, 125^ using the default classic FPKM normalization method and the default pooled dispersion model (Benjamini-Hochberg correction; significantly differentially expressed genes (DEGs) had q-value ≤ 0.05). Venn diagrams were generated using Vennt^126^. To further investigate the function of the DEGs, representative protein sequences (the longest sequence if there were splice variants) of every known honey bee gene were blasted against the *D*. *melanogaster* protein database via reciprocal BLAST+^127^ to identify fruit fly orthologs and homologs of the honey bee genes because there is a greater amount of gene ontology information for *D*. *melanogaster* genes compared to *Apis mellifera* genes. The honey bee genome encodes approximately 15,000 genes of which 13,592 genes are mapped and provided in the Amel4.5 genome annotation^128^. We annotated 8,944 genes (~66%) as homologs (of which 7,006 were reciprocal best hits or orthologs) to genes encoded by the fruit fly *D*. *melanogaster* genome, which encodes ~13,600 genes^128, 129^. Biological processes (BP) functional enrichment analysis was performed with DAVID^130^. Gene ontology and biological processes (BP-FAT) enrichment analysis was performed with DAVID^130^.

### Comparative analysis of DEGs in virus-infected bees

To identify the shared and unique DEGs in virus-infected bees, we compared our dataset to other studies that examined gene expression in virus-infected bees. Genes that were differentially expressed in SINV-GFP infected bees 72 hpi were compared to other studies that examined gene expression in virus-infected bees: symptomatic IAPV-fed bees^24^; SBV and DWV-infected bees^25^; adult honey bees naturally infected with IAPV^9^; and a common DEG list that was compiled from 19 gene expression data sets including *Varroa destructor*-parasitized and virus-infected bees^92^. We used NCBI Entrez Gene ID as a common identifier because DEG lists were generated using different technologies and versions of the *Apis mellifera* genome and transcriptome (Supplementary Table S14). DEGs were compared via Venn diagram analysis^131^ (Supplementary Table S15). Pairwise comparisons between studies identified shared DEGs and the statistical significance of gene overlap was assessed using hypergeometric tests^132^ (Supplementary Table S15).

### Identification of previously unrecognized honey bee transcript

RNASeq analysis determined that reads aligning to LOC725387 were more abundant in virus-infected bees. To identify the gene or genes encoded by these differentially expressed reads, the consensus nucleotide sequence was used to query the NCBI Nucleotide collection (nr/nt) and *A*. *mellifera* databases using blastn^133^, Sanger sequencing was performed to verify transcript sequence and length, and the results were evaluated using Geneious^134^. Together, these analyses revealed that we identified a previously unrecognized transcript, *A*. *mellifera* probable cyclin-dependent serine/threonine-protein kinase (MF116383, 5,158 nt), which is longer than the originally annotated *A*. *mellifera* probable serine/threonine-protein kinase clkA (LOC725387, XM_001121241.4, 1,403 nt).

In brief, we utilized LOC725387 RNASeq consensus sequence to query the NCBI Nucleotide nr/nt data base and identified an *A*. *cerana* transcript annotated as a probable cyclin-dependent serine/threonine-protein kinase DDB_G0292550 (LOC107994302, XM_017051141.1) as the top blastn result, which contained 95% of the submitted sequence and shared 91% identity (E-value = 0, 95% query coverage, 91% identity, 1–6% gaps); additional top blastn hits included *A*. *dorsata* GATA zinc finger domain-containing protein 14-like. When the LOC725387 RNASeq consensus sequence was used to query the *A*. *mellifera* database, the top blastn result only covered 24% of the query sequence (i.e., *A*. *mellifera* probable serine/threonine-protein kinase clkA, XM_001121241.4; E-value = 0, 24% query coverage, 99% identity, 0% gaps). To further characterize the LOC725387 transcript, we Sanger sequenced 5,027 nts (~2–3× coverage) and obtained the most 5′ end of this transcript from RNASeq data (131 bp, >2,000× coverage) (Supplementary Table S1 and Fig. S9). Together nucleotide and amino acid alignments indicate the RNAseq reads aligning to LOC725387 are most similar to a computationally predicted *A*. *cerana* cyclin-dependent serine/threonine-protein kinase DDB_G0292550 (Supplementary Fig. S9). Therefore, we refer to the gene identified herein as *A*. *mellifera probable cyclin*-*dependent serine/threonine*-*protein kinase* (Supplementary Fig. S9) and submitted the sequence of this transcript to NCBI (MF116383).

### Data Availability

The majority of the data generated or analyzed during this study are included in this published article and its Supplementary Information files (available online), additional data is available from the corresponding author upon request, and sequence data may be accessed from the NCBI Sequence Read Archive (accession number SRP101337).

## Electronic supplementary material

Supplementary Information

Supplementary Tables
