# Supplementary material for: Virus and dsRNA-triggered transcriptional responses reveal key components of honey bee antiviral defense
Source: Sci Rep. 2017 Jul 25;7:6448. doi: 10.1038/s41598-017-06623-z (PMC5526946; doi:10.1038/s41598-017-06623-z)
Supplement: Supplementary file 1 — Supplementary Information [file 41598_2017_6623_MOESM1_ESM.pdf]

Virus and dsRNA-triggered transcriptional responses reveal key components of honey bee antiviral defense

Laura M. Brutscher[1,2,3], Katie F. Daughenbaugh[1,3], and Michelle L. Flenniken[*,1,2,3]
[1]Department of Plant Sciences and Plant Pathology, [2]Department of Microbiology and Immunology, [3]Pollinator Health Center, Montana State University, Bozeman, MT, USA
*corresponding author: michelle.flenniken@montana.edu

S1 Fig. Fluorescence microscopy of virus-infected bees indicates that dsRNA reduced virus abundance.

Fluorescence microscope images of the abdomens of virus-infected bees indicate that dsRNA reduced virus abundance. Pictured are representative images of mock-infected bees, virus-infected bees, virus-infected and virus-specific dsRNA (sp-dsRNA)-treated bees, and virus-infected and non-specific dsRNA (ns-dsRNA)-treated bees at 72 hpi using fluorescence (top row) and light microscopy (bottom row). Virus-infected bees that were treated with either sp-dsRNA or ns-dsRNA exhibited decreased fluorescence as compared to virus-infected bees that were not treated with dsRNA.

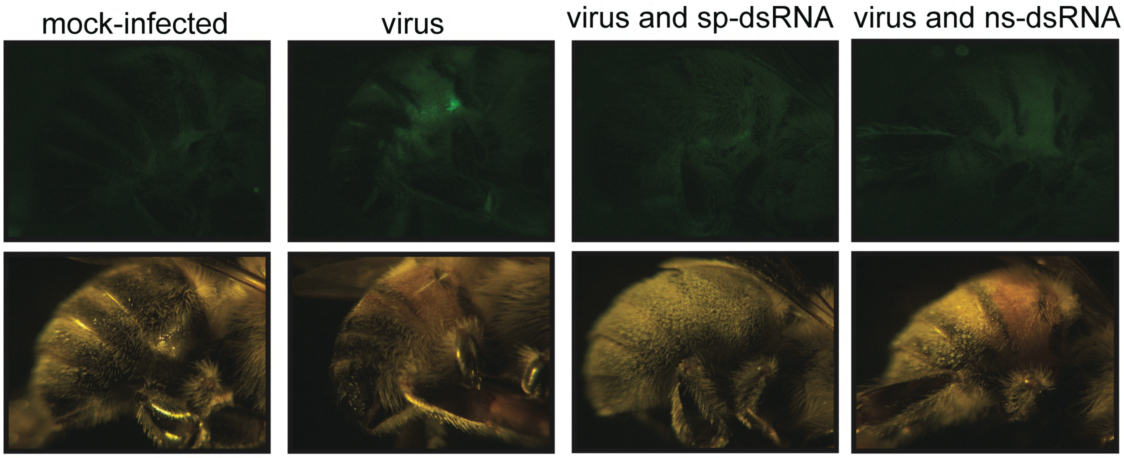

S2 Fig. Viral GFP production was reduced in dsRNA treated honey bees

Western blot analysis of individual bee lysates 72 hours post infection (hpi) using α-GFP and α-β-actin antibodies. (A) Bees treated with dsRNA, sp-dsRNA (lanes 8-12) and (B) ns-dsRNA (lanes 13-17), exhibited reduced Sindbis-GFP as compared to (A) virus infected bees (lanes 1-5) and (B) virus and NTP-treated bees (lanes 20-24); (-) denotes pooled samples from mock-infected bees and (+) denotes pooled samples from virus-infected positive control samples. (B) ImageJ was utilized to quantify the pixel count in each band, and the GFP: β-actin ratio was calculated for each sample and plotted on a box-and-whisker; p-values were assessed via Wilcoxon rank-sum tests. The median GFP to actin ratio was 1.0 for virus-infected bees, whereas the median ratio in bees that were co-injected with sp-dsRNA was 0.3 ($p = 0.03$). Similarly, co-injection of virus and ns-dsRNA reduced the relative production of SINV-GFP as compared to co-injected with virus and NTPs, with median ratios of 0.5 versus 1.1 ($p = 0.01$). The sample size for each treatment group in this representative Western blot was five, although numerous other samples were analyzed via Western blot.

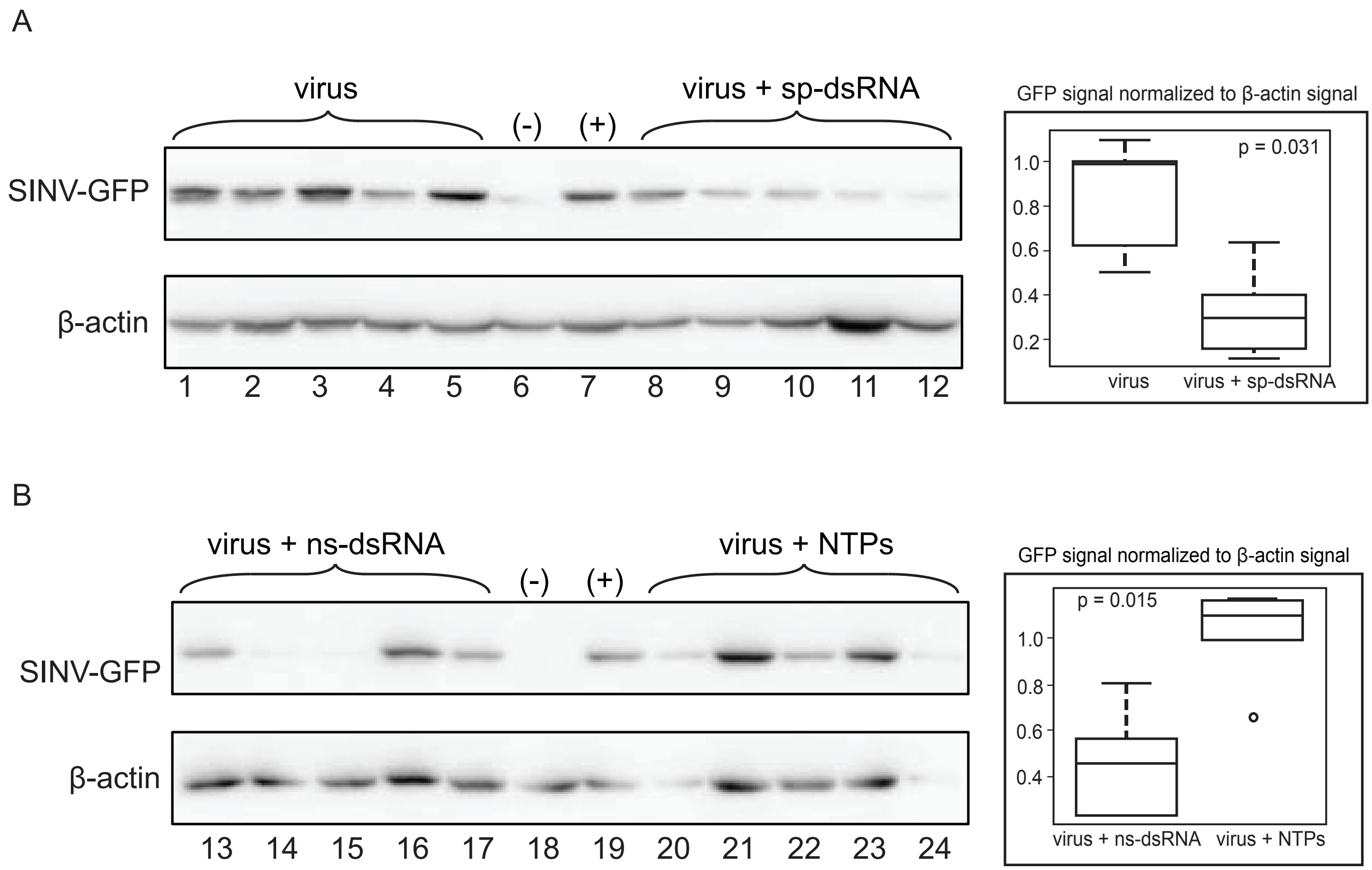

S3 Fig. Virus abundance in honey bees increased with time post-infection

Relative virus RNA abundance (including both virus genomes and transcripts) was assessed in the abdomens of virus-infected bees 6, 48, and 72 hpi (n=10). Little to no virus was detected in virus-infected bees 6 hpi. Relative virus abundance increased as time post-infection increased. Relative virus abundance was assessed using qPCR and ΔΔCT analysis; *Am rpl8* was used as the house keeping gene and ΔΔCT data was normalized to virus-infected bees at 72 hpi. The bars represent standard error of the mean.

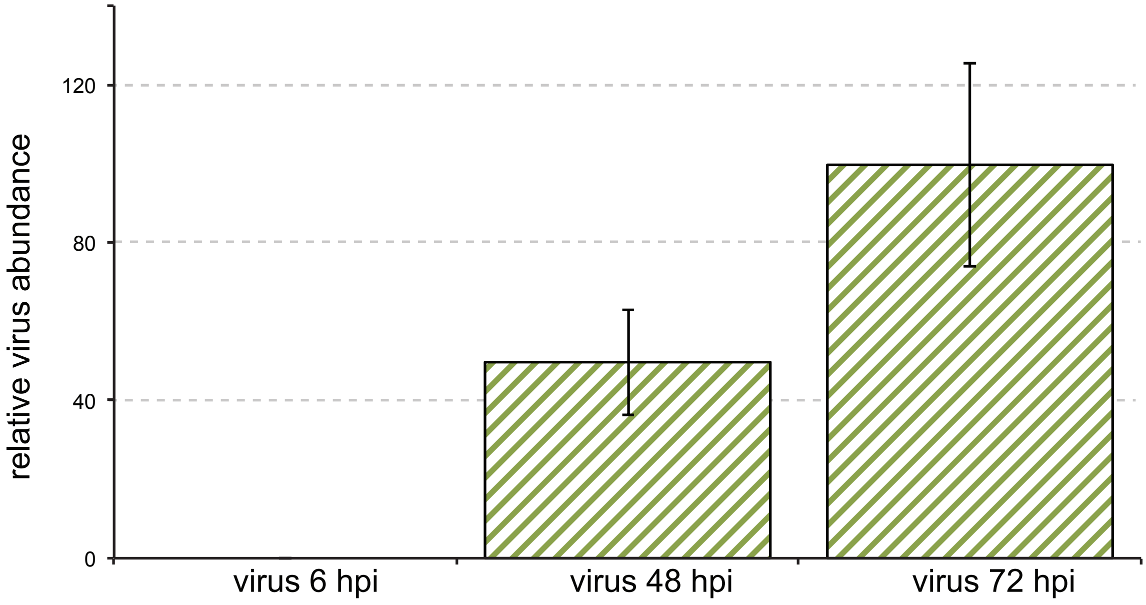

S4 Fig. Relative virus RNA abundance was reduced in dsRNA-treated bees, as compared to virus-infected bees, in three biological replicates

The abdomens of bees (A) 48 and (B) 72 hours post-infection (hpi) have reduced relative virus abundance when treated with dsRNA, whether it is sequence-specific to the virus (sp-dsRNA, dotted purple) or nonspecific (ns-dsRNA, checkered blue). This result was consistent in three different biological replicates, including bees from different colonies. Treatment with NTPs (wavy orange lines) did not result in different relative virus abundance. Biological replicate 1 (rep 1) includes bees that were utilized for RNAseq analysis for which their relative virus abundance is also shown in Fig 2. Biological replicates 2 and 3 were bees collected from different colonies and likely represent bees with different genetic compositions. Relative virus abundance was assessed using qPCR and ΔΔCT analysis, using *Am rpl8* as the house keeping gene; statistical differences between treatment and virus-infected bees (n=10) were performed using student's t-test, *p< 0.05, **p< 0.005. The bars represent standard error of the mean.

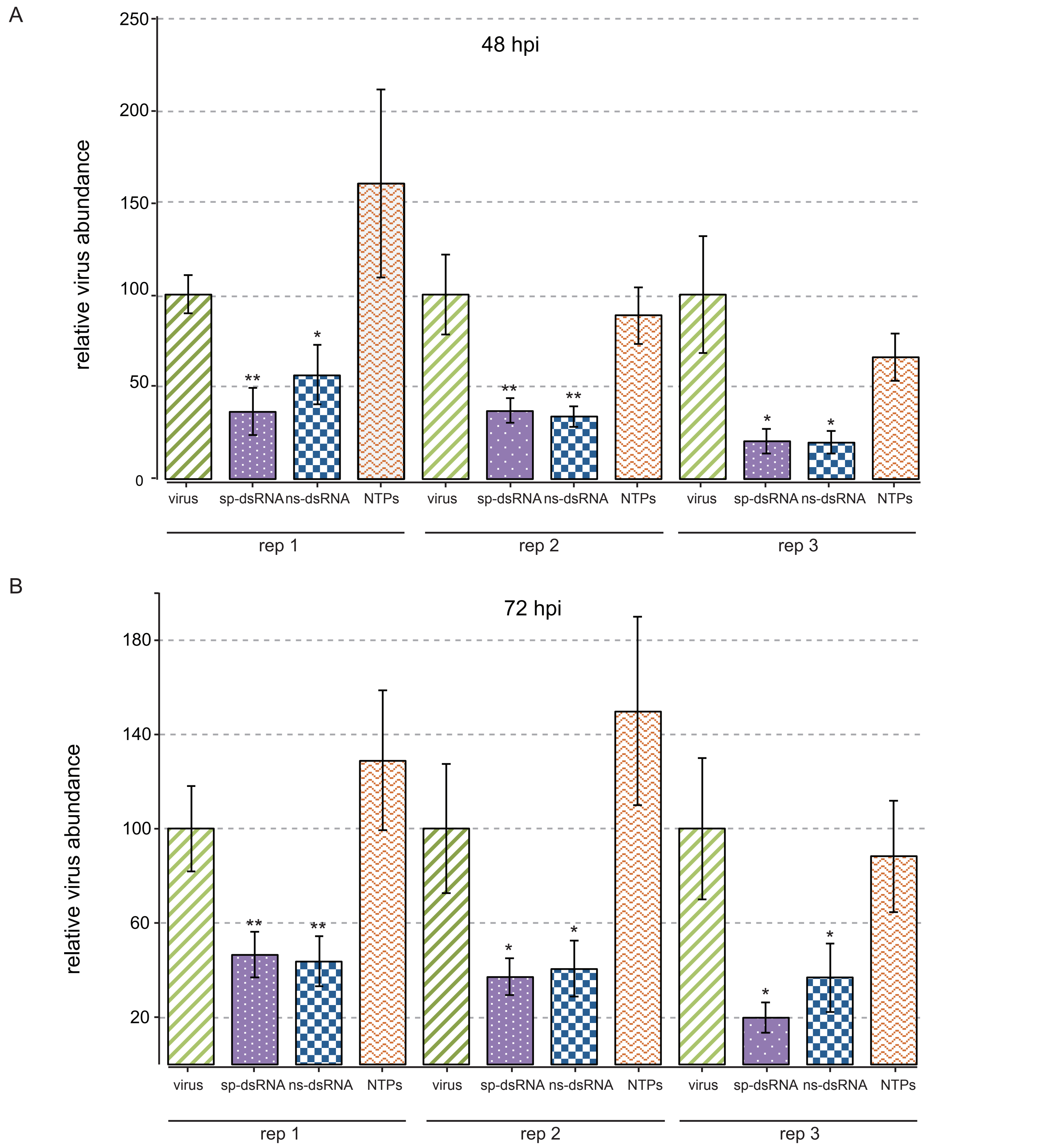

S5 Fig. Venn diagram of shared and unique DEGs in virus-infected and/or dsRNA-treated bees

Venn diagrams were utilized to identify shared and unique differentially expressed genes (DEGs) among treatment groups within each time point post-infection. By identifying DEGs that were shared by virus-infected bees (green), dsRNA-treated bees (orange), bees treated with both virus and virus-specific dsRNA (sp-dsRNA, blue), and bees treated with virus and nonspecific dsRNA (ns-dsRNA, purple), we could identify the number of genes that are induced by biologically relevant levels of dsRNA at (A) 6 hpi, (B) 48 hpi, and (C) 72 hpi, as well as identify DEGs specific to each treatment or each specific comparison of treatments. (D) The shared DEGs in all virus-infected bees at 48 hpi and 72 hpi were too large to visualize in Venn diagrams, therefore the number of shared DEGs were expressed in table format. In order to identify genes that may contribute to enhanced antiviral defense in dsRNA-treated bees (Fig 2), the shared genes with increased expression in all virus-infected and dsRNA treated bees, but not shared with the bees that were only infected with virus (five genes), were assessed. Five genes exhibited increased expression in virus-infected and dsRNA treated bees, but not in bees only infected with virus. There were two genes with decreased expression in all virus and dsRNA treated bees, but not in virus treated bees. Lists of the specific shared genes in all Venn diagrams (A-F) are provided in Tables S9-S13.

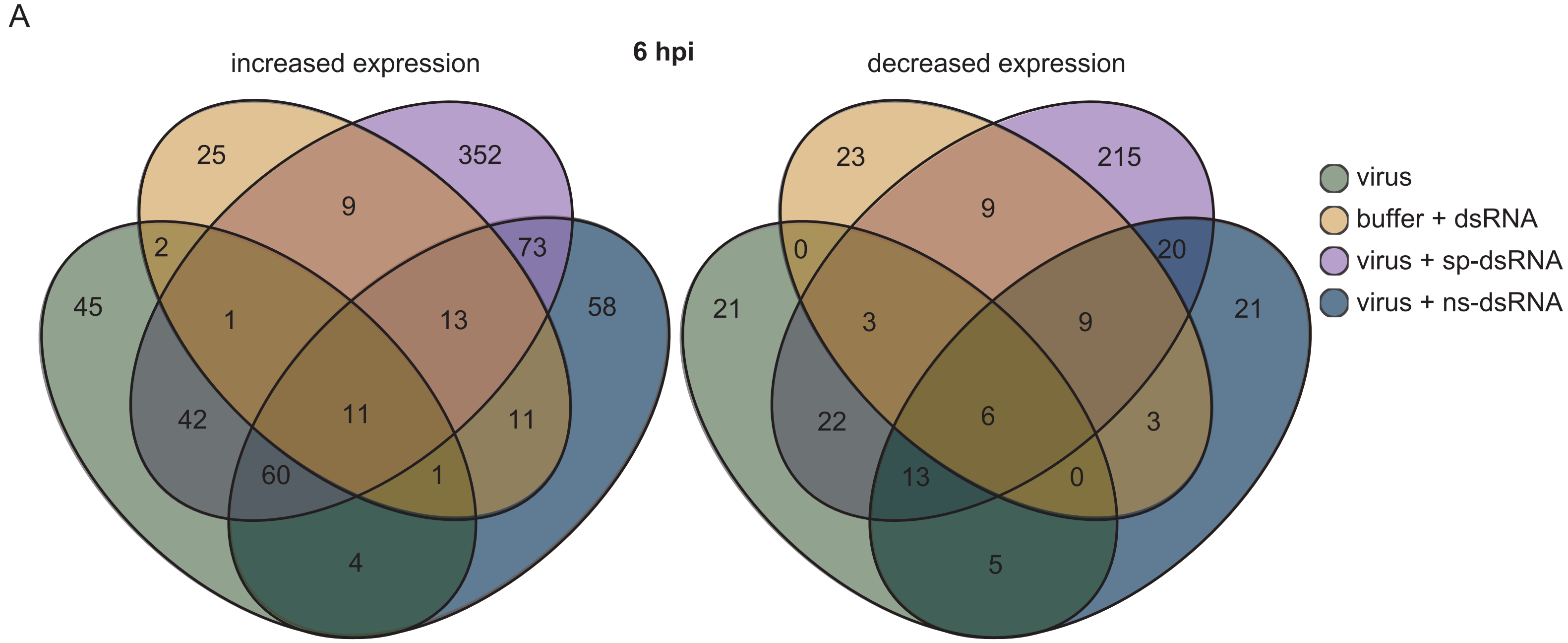

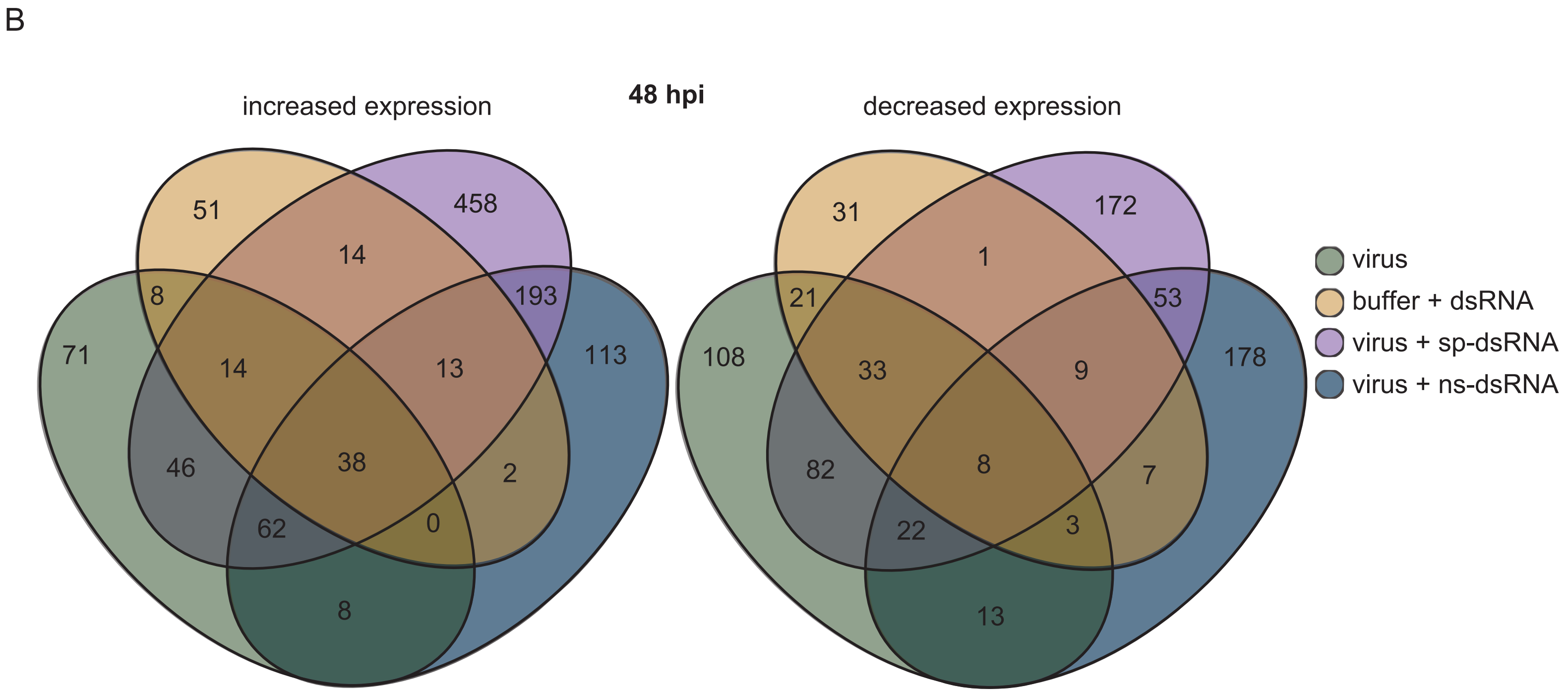

C

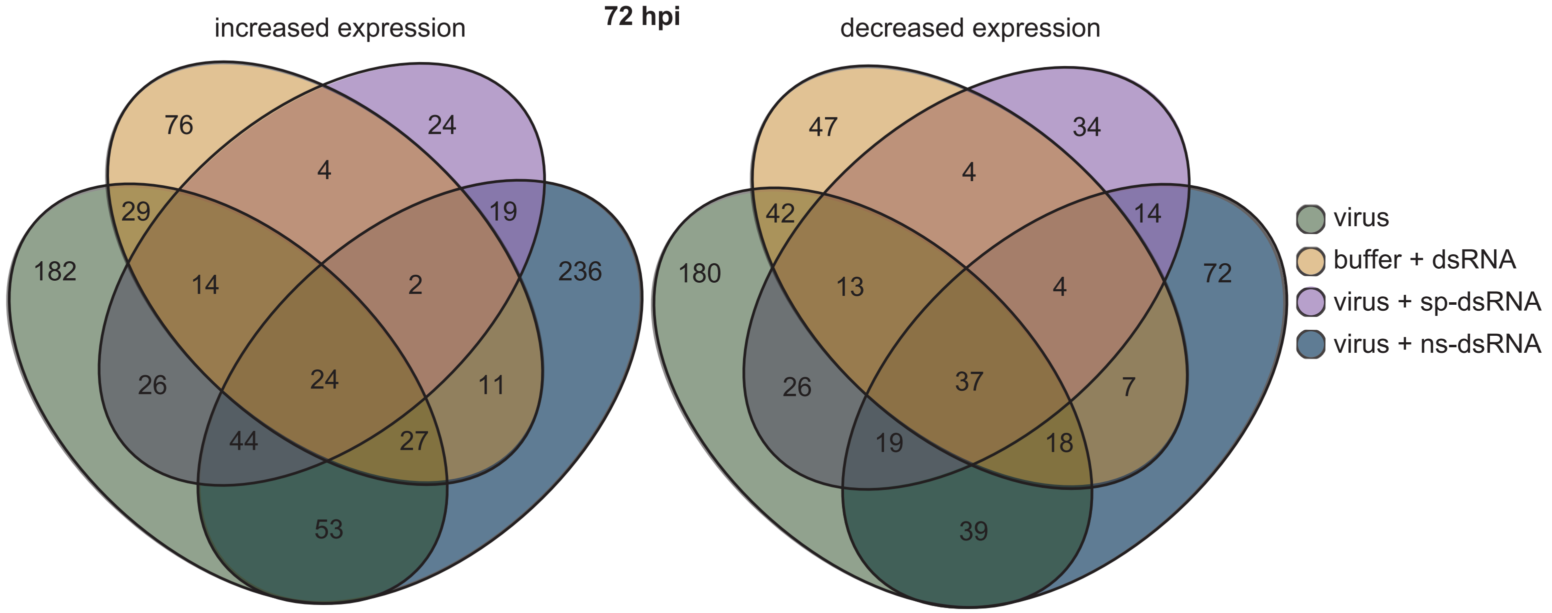

D

increased expression

virus 48 hpi	virus + sp-dsRNA 48 hpi	virus + ns-dsRNA 48 hpi	virus 72 hpi	virus + sp-dsRNA 72 hpi	virus + ns-dsRNA 72 hpi
26	26	26	26	26	26
	5	5		5	5
60					
	356				
		92			
			176		
				18	
					79

decreased expression

virus 48 hpi	virus + sp-dsRNA 48 hpi	virus + ns-dsRNA 48 hpi	virus 72 hpi	virus + sp-dsRNA 72 hpi	virus + ns-dsRNA 72 hpi
7	7	7	7	7	7
	2	2		2	2
90					
	158				
		143			
			163		
				30	
					54

S6 Fig. Gene ontology enrichment analysis of DEGs determined that biological processes including phosphorylation morphogenesis, transcription, and development were differentially regulated in virus-infected bees 6 hpi

Gene ontology (GO) enrichment analysis was performed using DAVID to identify the top biological processes (BP) perturbed by virus infection at 6 hpi, 48, and 72 hpi. (A) DEGs at 6 hpi were enriched for several GO terms, including phosphorylation (14 genes), pattern specification (12 genes), imaginal disc development (12 genes), and cell death (5 genes). (B) Virus-infected bees 48 hpi exhibited DEGs enriched for functions including morphogenesis (20 genes), cell adhesion (10 genes), and immune response (8 genes). (C) Virus-infected bees 72 hpi exhibited DEGs enriched for functions including transcriptional regulation, cell morphogenesis, gene silencing, and protein folding. *Similar to 48 and 72 hpi, several genes involved in transcriptional regulation were also differentially expressed at 6 hpi and were worth noting, but was not significantly enriched. DAVID gene enrichment p-values are listed to the right of each graph.

A

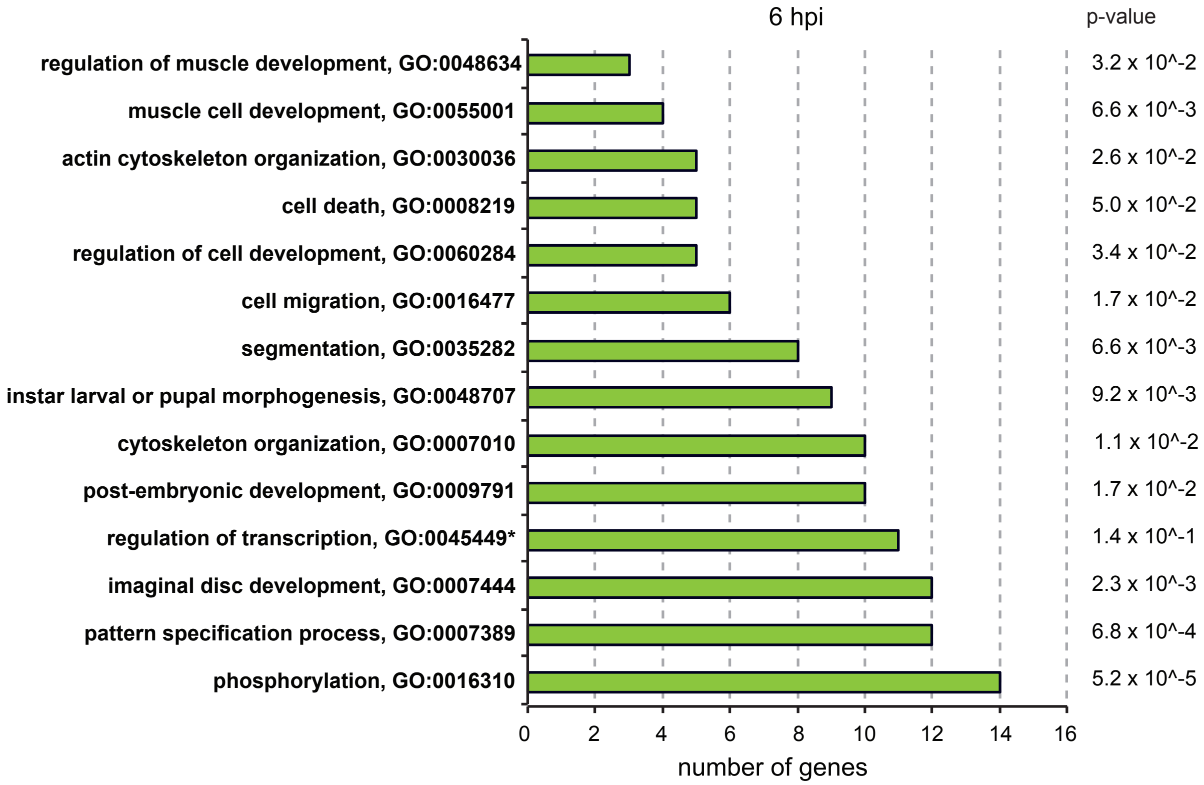

B

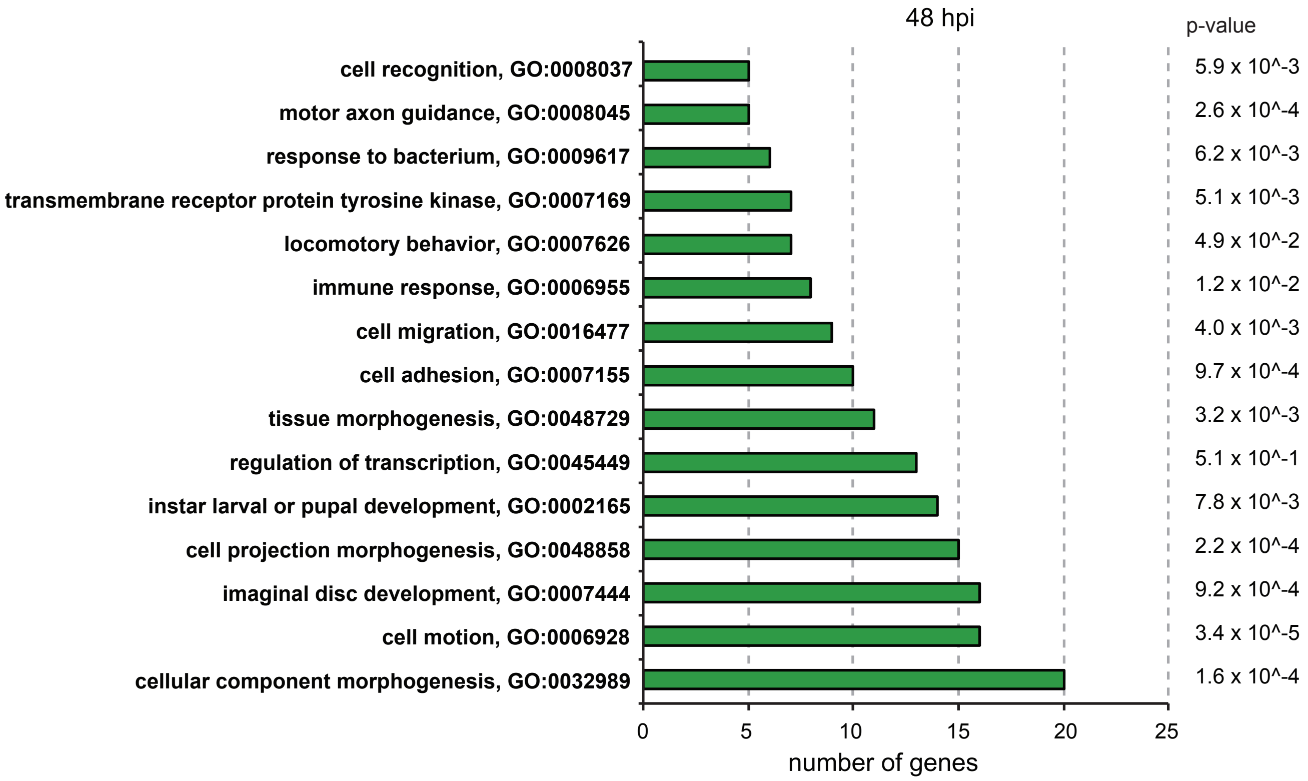

48 hpi
p-value
cell recognition, GO:0008037
motor axon guidance, GO:0008045
response to bacterium, GO:0009617
transmembrane receptor protein tyrosine kinase, GO:0007169
locomotory behavior, GO:0007626
immune response, GO:0006955
cell migration, GO:0016477
cell adhesion, GO:0007155
tissue morphogenesis, GO:0048729
regulation of transcription, GO:0045449
instar larval or pupal development, GO:0002165
cell projection morphogenesis, GO:0048858
imaginal disc development, GO:0007444
cell motion, GO:0006928
cellular component morphogenesis, GO:0032989
5.9 x 10^-3
2.6 x 10^-4
6.2 x 10^-3
5.1 x 10^-3
4.9 x 10^-2
1.2 x 10^-2
4.0 x 10^-3
9.7 x 10^-4
3.2 x 10^-3
5.1 x 10^-1
7.8 x 10^-3
2.2 x 10^-4
9.2 x 10^-4
3.4 x 10^-5
1.6 x 10^-4
0
5
10
15
20
25
number of genes

C

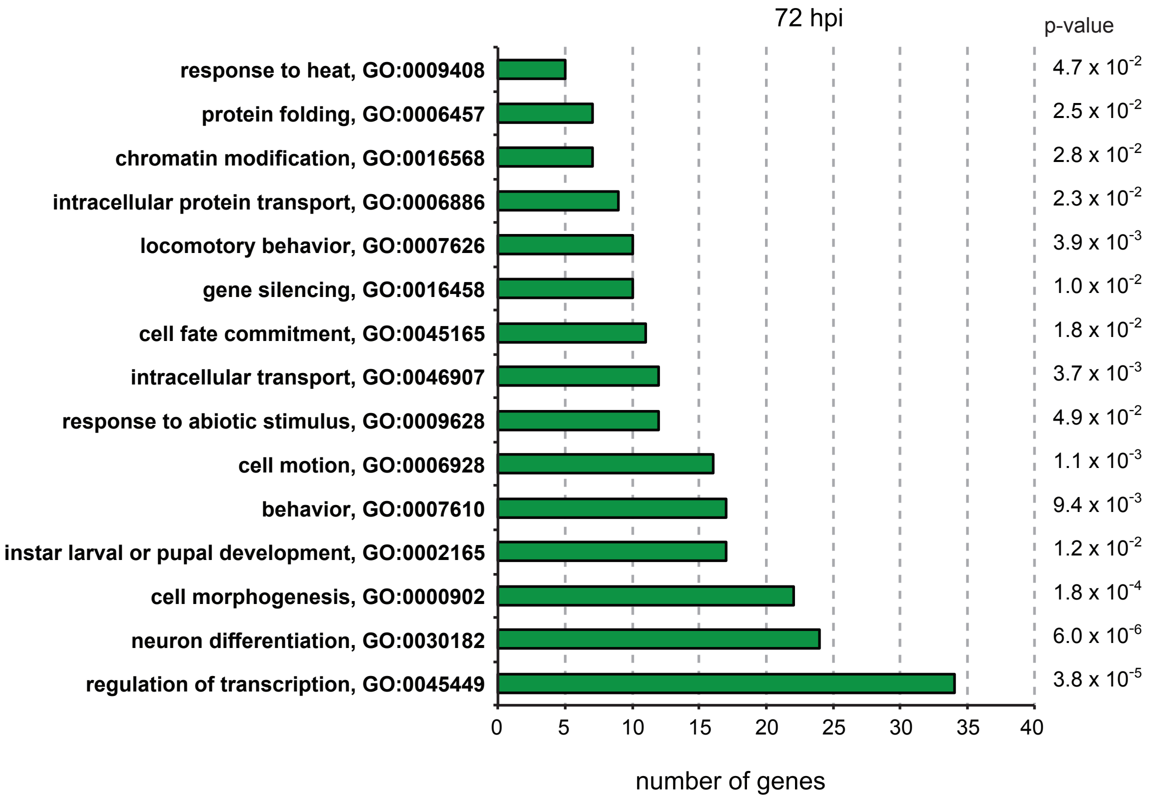

72 hpi
p-value
response to heat, GO:0009408
protein folding, GO:0006457
chromatin modification, GO:0016568
intracellular protein transport, GO:0006886
locomotory behavior, GO:0007626
gene silencing, GO:0016458
cell fate commitment, GO:0045165
intracellular transport, GO:0046907
response to abiotic stimulus, GO:0009628
cell motion, GO:0006928
behavior, GO:0007610
instar larval or pupal development, GO:0002165
cell morphogenesis, GO:0000902
neuron differentiation, GO:0030182
regulation of transcription, GO:0045449
4.7×10^{-2}
2.5×10^{-2}
2.8×10^{-2}
2.3×10^{-2}
3.9×10^{-3}
1.0×10^{-2}
1.8×10^{-2}
3.7×10^{-3}
4.9×10^{-2}
1.1×10^{-3}
9.4×10^{-3}
1.2×10^{-2}
1.8×10^{-4}
6.0×10^{-6}
3.8×10^{-5}
0
5
10
15
20
25
30
35
40
number of genes

S7 Fig. qPCR analysis of a subset of DEGs confirms RNAseq results in bees at 48 hpi

Transcriptome level sequencing (RNAseq) identified hundreds of honey bee (*Apis mellifera*) genes that exhibited differential expression in the context of virus infection and/or dsRNA-treatment, and qPCR was utilized to validate RNAseq results of 10 genes that exhibited increased expression in bees 48 hpi (q < 0.05 after BH correction, Tables S5 and S6). The nine DEGs exhibited increased expression in virus-infected bees (green stripes) and virus and dsRNA treated bees (sp- and ns dsRNA, dotted purple and checkered blue) via qPCR analysis including *hsp90*, *abaecin*,probable *cyclin-dependent kinase*, *ago2*, dicer, *igfn3-10*, *orb-2-like*, *mfs-type transporter*, and *fam102b*; whereas *jra* did not, which was expected based on RNAseq analysis. Two DEGs, *jra*, and *fam102b*, exhibited increased expression in the dsRNA (dotted orange) bees, which was expected. Relative gene transcript abundance was assessed using qPCR and ΔΔCT analysis, using *Am rpl8* as the house keeping gene; expression in each treatment group was compared to mock-infected controls. Thirteen genes were also qPCR validated in bees at 72 hpi (Fig 4). Statistical differences in gene expression between mock-infected and treatment bees (n=5) were performed using Welch's two sample t-tests, *p< 0.05, **p< 0.005. The bars represent standard error of the mean.

A

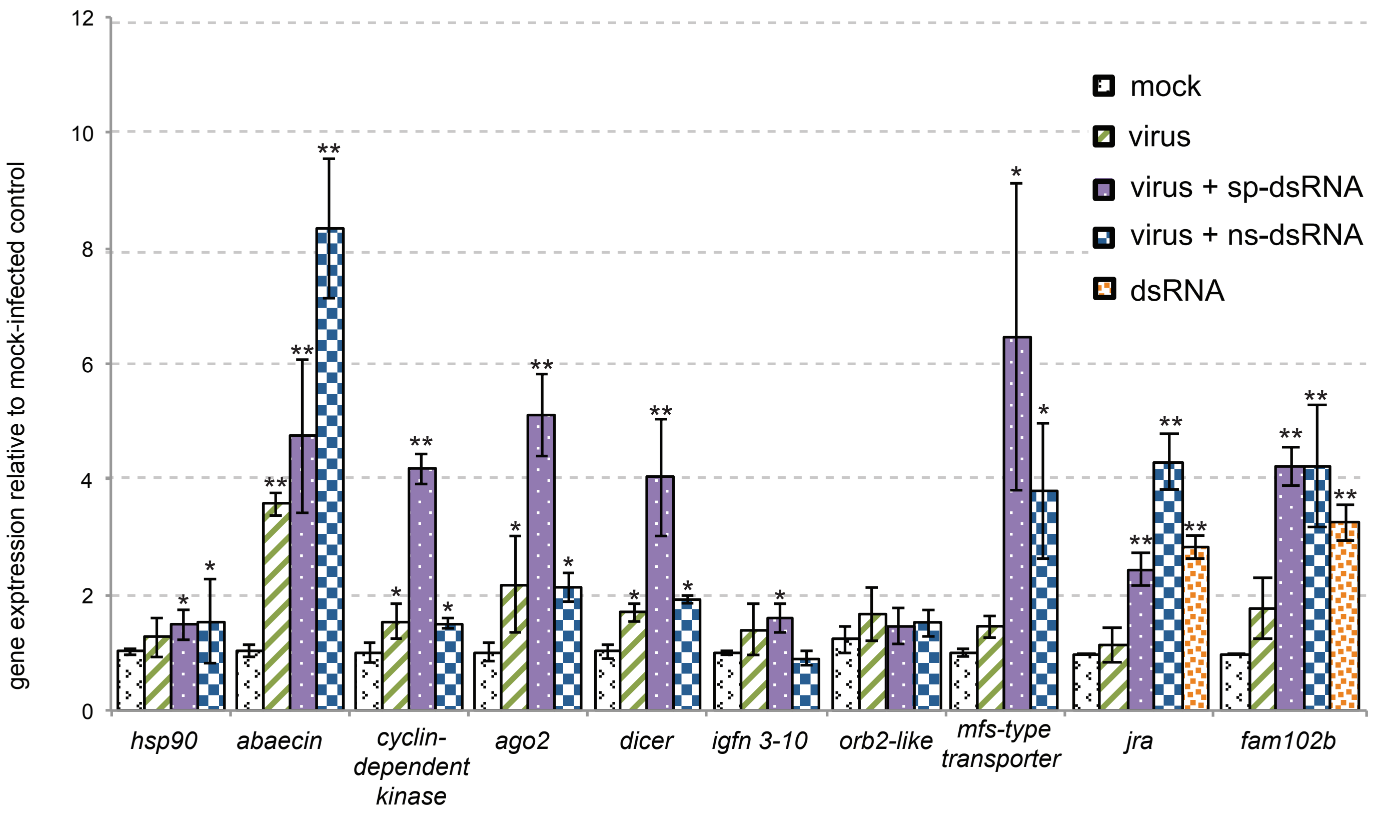

A

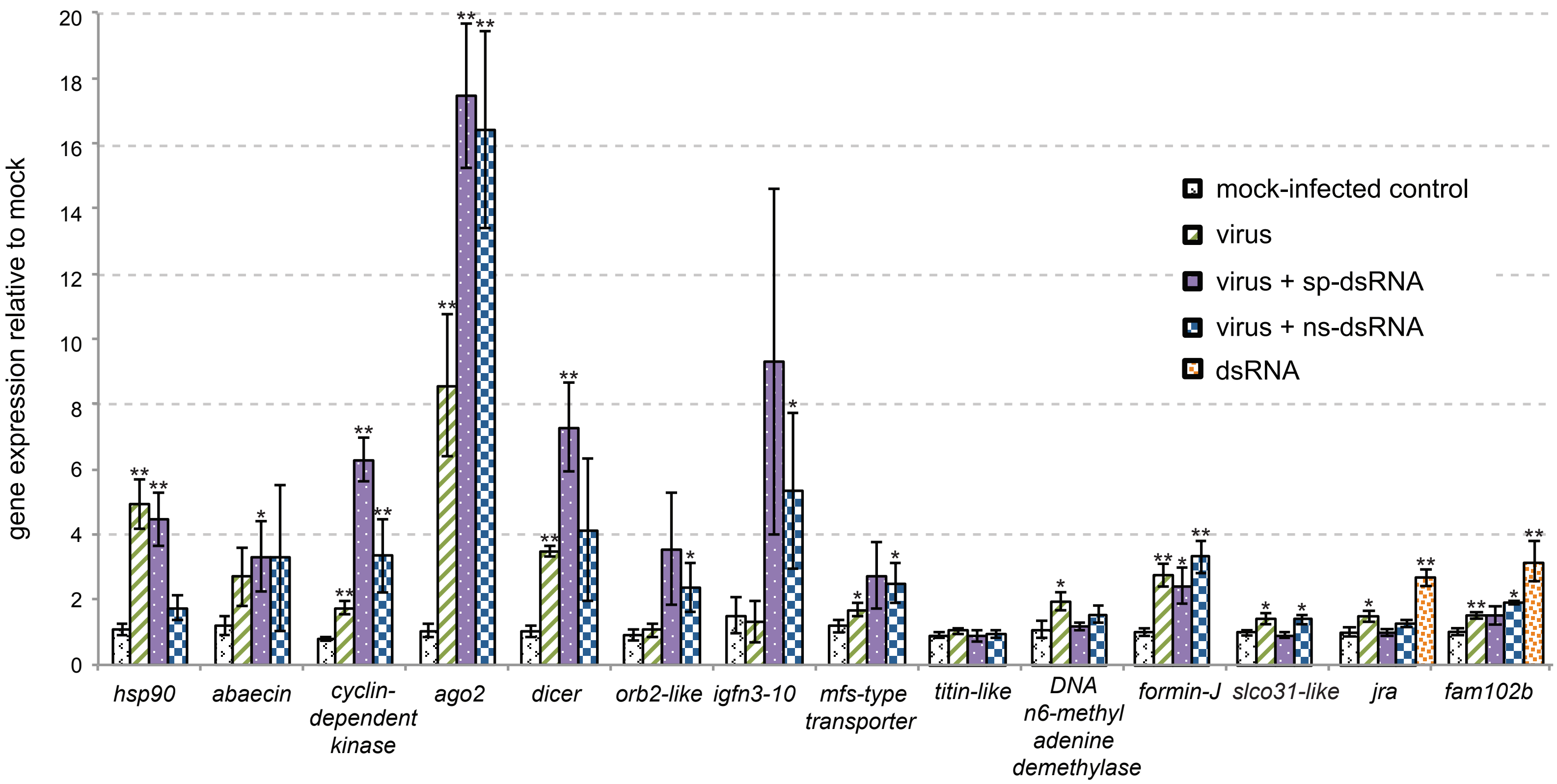

gene expression relative to mock
20
18
16
14
12
10
8
6
4
2
0
mock-infected control
virus
virus + sp-dsRNA
virus + ns-dsRNA
dsRNA
hsp90
abaecin
cyclin-dependent kinase
ago2
dicer
orb2-like
igfn3-10
mfs-type transporter
titin-like
DNA n6-methyl adenine demethylase
formin-J
slco31-like
jra
fam102b

S8 Fig. qPCR analyses confirmed differential expression of putative antiviral genes in bees from different genetic backgrounds

Transcriptome analysis (RNAseq) of virus-infected and/or dsRNA-treated bees identified numerous candidate honey bee antiviral genes that exhibited greater expression as compared to mock-infected controls (q-value < 0.05 after BH correction), including the genes (A) *hsp90*, (B) *cyclin-dependent serine/threonine kinase*, (C) *ago2*, (D) *dicer*, (E) *mfs-transporter*, (F) *formin-j*, (G) *tet-2*, (H) *orb-2*, (I) *3a1-like*, (J) and *igfn 3-10*. qPCR analysis was performed for these genes on the bees that were sequenced (rep 1). (A-J) In order to confirm that these genes consistently exhibit increased expression in response to virus infection in honey bees with different genetic backgrounds, qPCR was performed on pooled samples (5 bees per pool) from virus-infected bees that were collected from different honey bee colonies (reps 2 and 3). (A-F) Several of these genes displayed increased expression via qPCR in all three biological replicates (A-D, G, J), but not as robustly, perhaps due to the fact that the samples were pooled as opposed to individually analyzed as in rep 1. (E, H, I) Other genes, displayed increased expression in 2 of the 3 biological replicates. (F) One gene, *igfn 3-10*, only exhibited increased expression in the sequenced bees (rep 1) and not in the other biological replicates (rep 2 and 3). Normalized gene transcript abundance was assessed using qPCR and ΔΔCT analysis using *Am rpl8* as the house keeping gene. The bars are standard error of the mean.

A

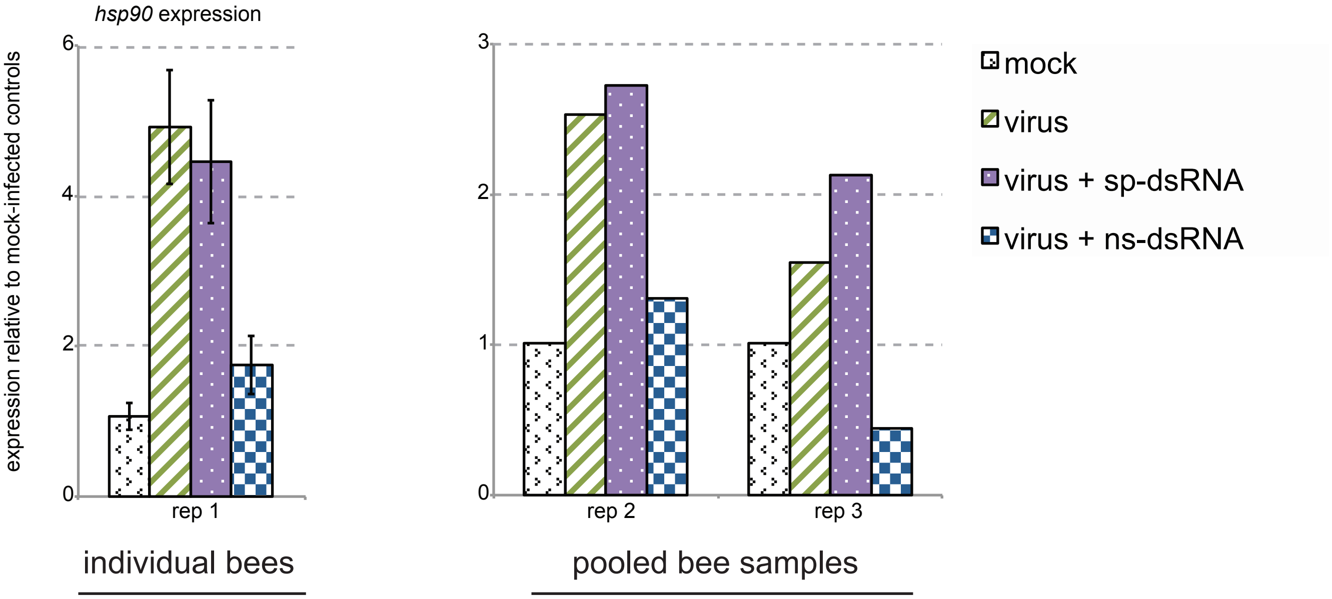

B

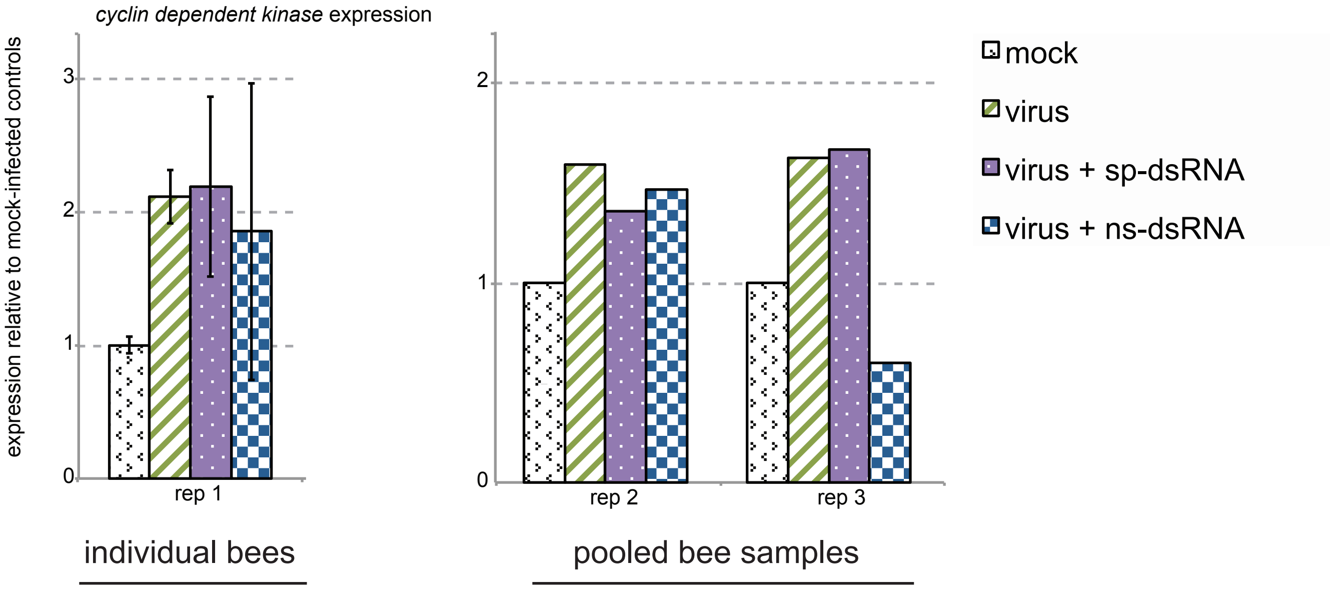

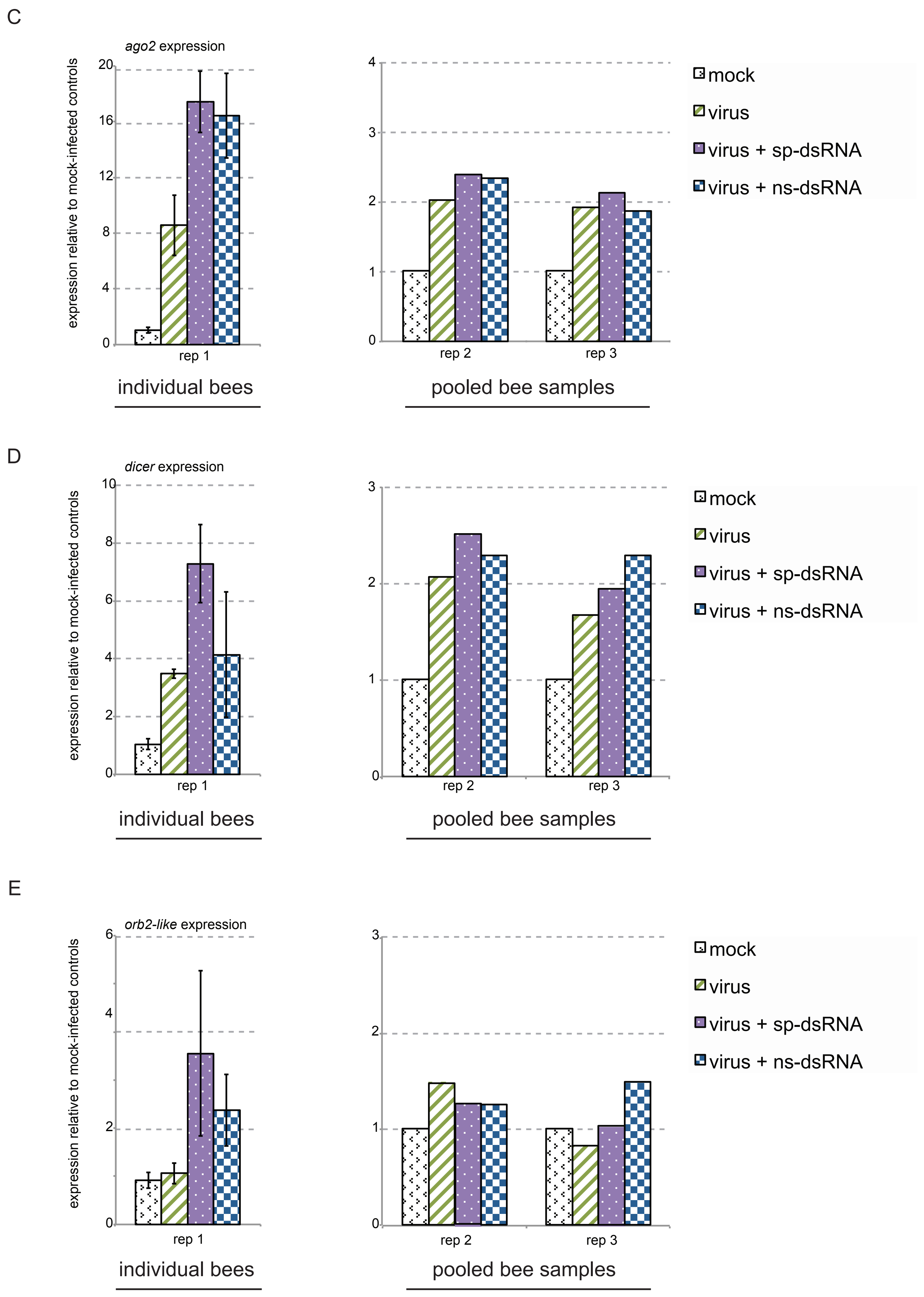
C
ago2 expression
expression relative to mock-infected controls
0
4
8
12
16
20
rep 1
individual bees
0
1
2
3
4
rep 2
rep 3
pooled bee samples
mock
virus
virus + sp-dsRNA
virus + ns-dsRNA
D
dicer expression
expression relative to mock-infected controls
0
2
4
6
8
10
rep 1
individual bees
0
1
2
3
rep 2
rep 3
pooled bee samples
mock
virus
virus + sp-dsRNA
virus + ns-dsRNA
E
orb2-like expression
expression relative to mock-infected controls
0
2
4
6
rep 1
individual bees
0
1
2
3
rep 2
rep 3
pooled bee samples
mock
virus
virus + sp-dsRNA
virus + ns-dsRNA

F

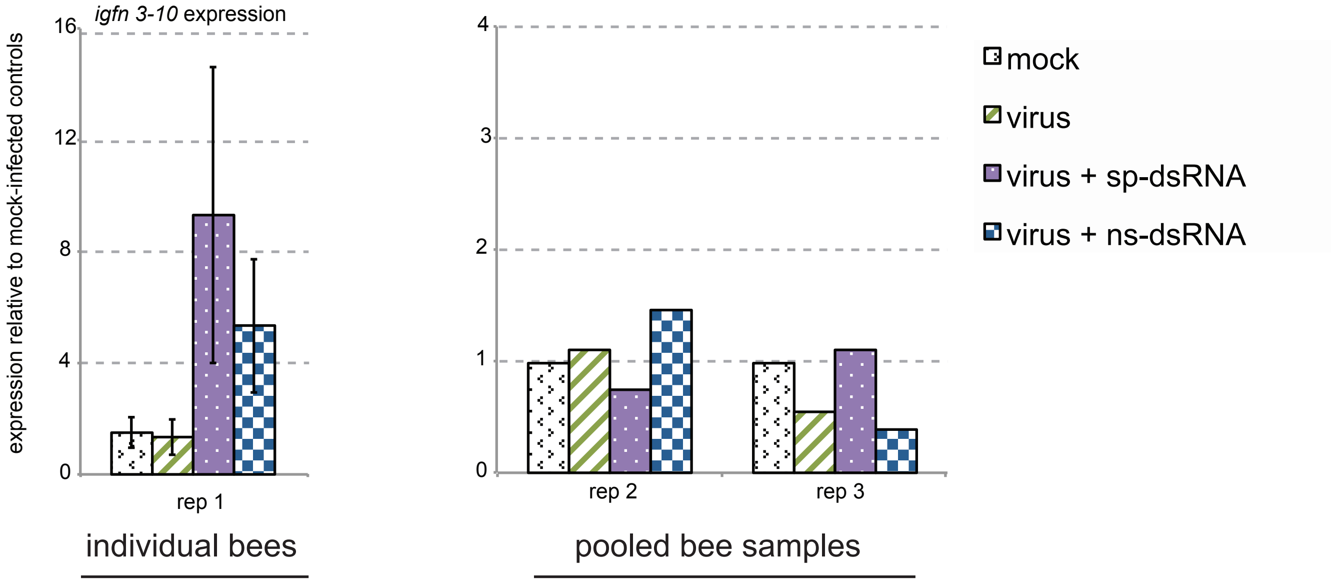
igfn 3-10 expression
expression relative to mock-infected controls
16
12
8
4
0
4
3
2
1
0
rep 1
rep 2
rep 3
individual bees
pooled bee samples
mock
virus
virus + sp-dsRNA
virus + ns-dsRNA

G

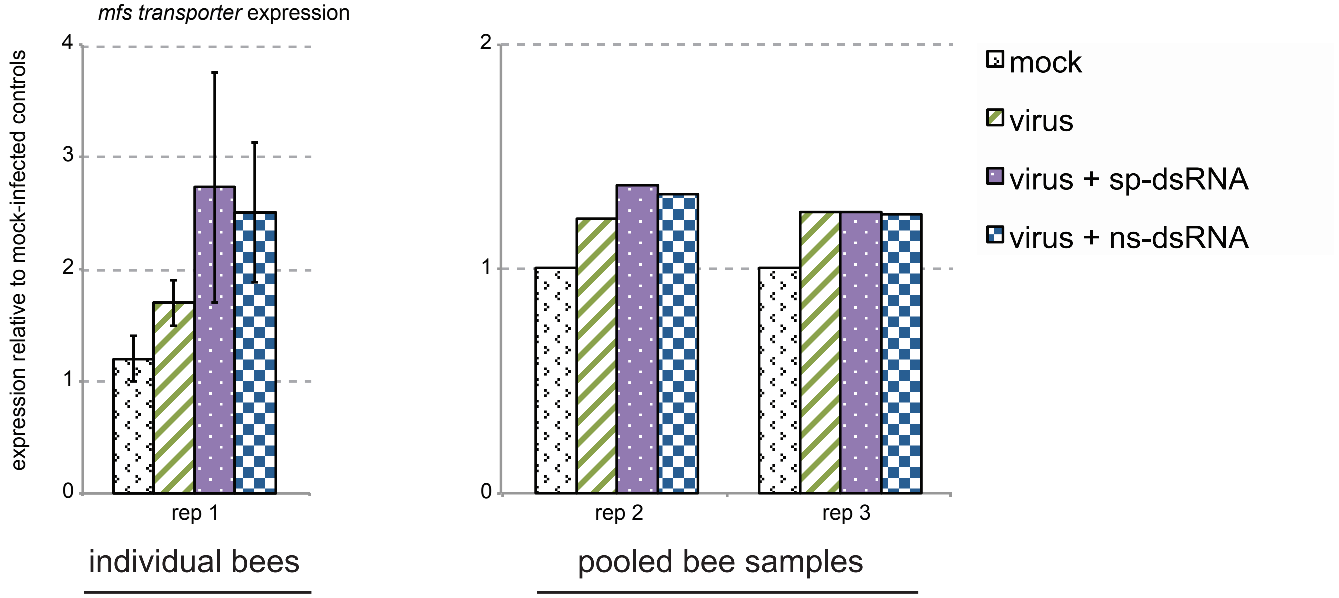
mfs transporter expression
expression relative to mock-infected controls
4
3
2
1
0
2
1
0
rep 1
rep 2
rep 3
individual bees
pooled bee samples
mock
virus
virus + sp-dsRNA
virus + ns-dsRNA

H

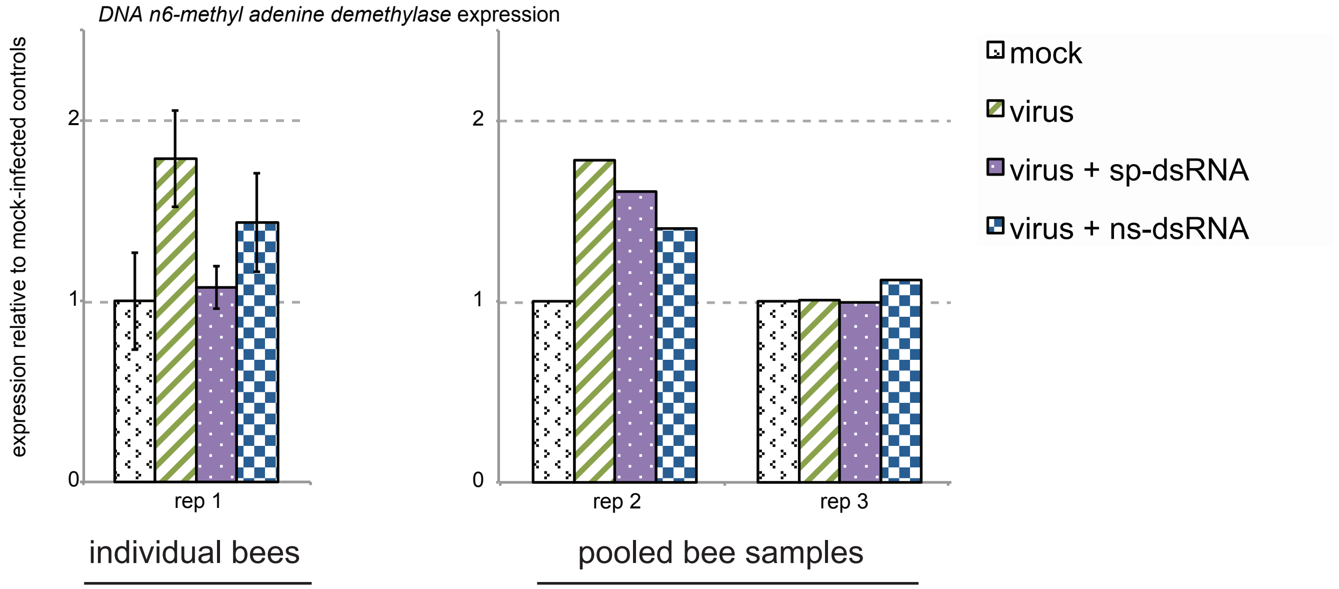
DNA n6-methyl adenine demethylase expression
expression relative to mock-infected controls
2
1
0
2
1
0
rep 1
rep 2
rep 3
individual bees
pooled bee samples
mock
virus
virus + sp-dsRNA
virus + ns-dsRNA

I

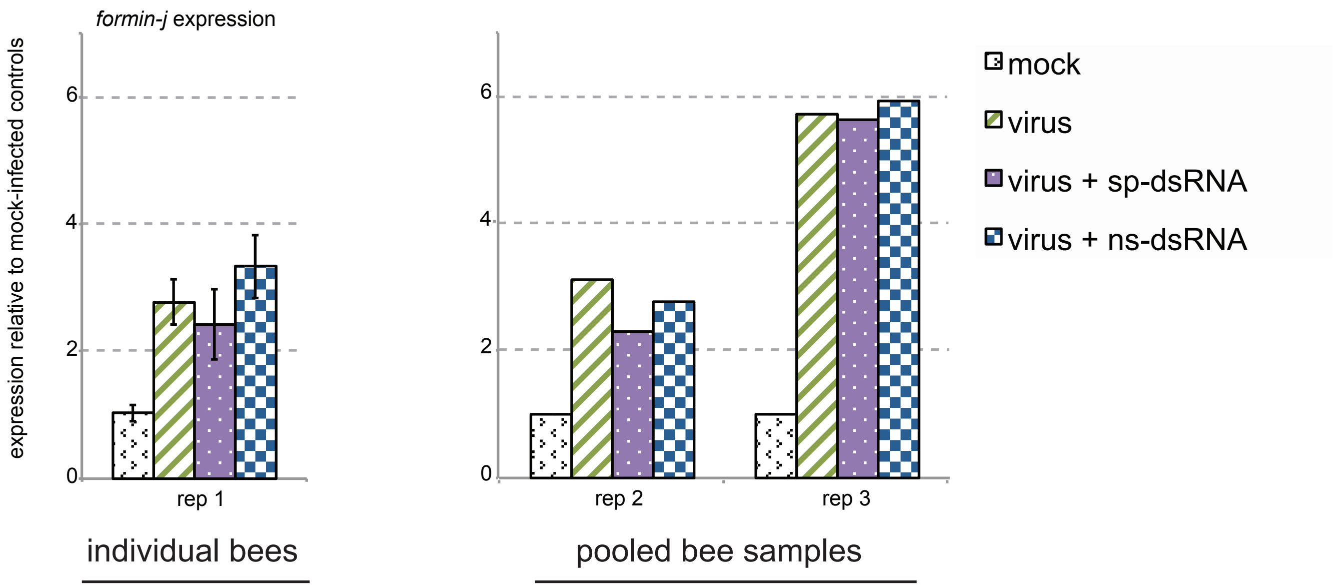
formin-j expression
expression relative to mock-infected controls
0
2
4
6
rep 1
individual bees
0
2
4
6
rep 2
rep 3
pooled bee samples
mock
virus
virus + sp-dsRNA
virus + ns-dsRNA

J

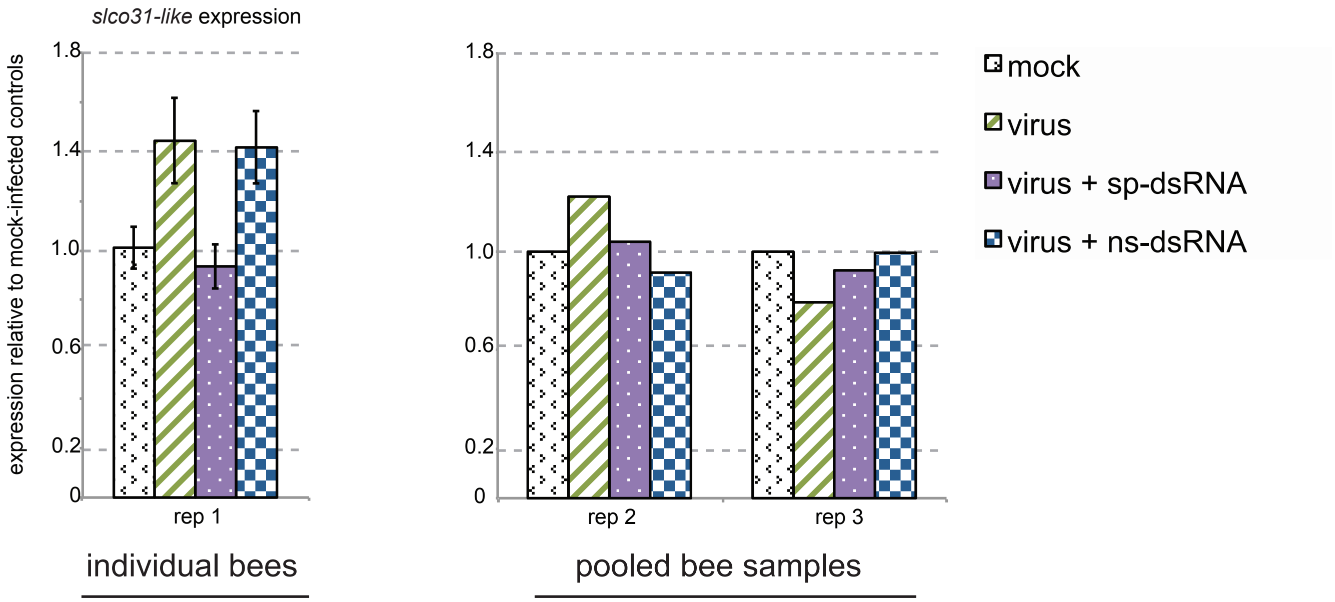
slco31-like expression
expression relative to mock-infected controls
1.8
1.4
1.0
0.6
0.2
0
rep 1
individual bees
1.8
1.4
1.0
0.6
0.2
0
rep 2
rep 3
pooled bee samples
mock
virus
virus + sp-dsRNA
virus + ns-dsRNA

S9 Fig. Identification of previously unrecognized honey bee transcript, *A. mellifera* probable cyclin-dependent serine/threonine-protein kinase transcript (MF116383)

RNASeq analysis determined that reads aligning to LOC725387 were more abundant in virus-infected bees. To identify the gene or genes encoded by these differentially expressed reads, the consensus nucleotide sequence was used to query the NCBI Nucleotide collection (nr/nt) and *A. mellifera* databases using blastn, Sanger sequencing was performed to verify transcript sequence and length, and the results were evaluated using Geneious.

(A) The updated LOC725387 *A. mellifera* probable cyclin-dependent serine/threonine-protein kinase transcript (5,158 nt, MF116383), *A. cerana* probable cyclin-dependent serine/threonine-protein kinase transcript (XM_017051141.1), *A. dorsata* GATA zinc finger domain-containing protein 14-like, transcript variant X1 (XM_006614476.1), and the original *A. mellifera* probable serine/threonine-protein kinase clkA transcript (XM_001121241.4) were aligned in Geneious (65% similarity Cost Matrix, 12 nt gap open penalty, 3 nt gap extension penalty, 2 refinement iterations). The updated *A. mellifera* transcript shared 85.2% nucleotide identity (4,271/5,021 nt) with entire *A. cerana* transcript. The previously annotated *A. mellifera* transcript (XM_001121241.4) is 1,587 nucleotides in length and 99.8% identical to the 5' end of the longer updated *A. mellifera* transcript (MF116383). There is mismatch between the updated *A. mellifera* transcript (MF116383) and the previously annotated *A. mellifera* transcript (XM_001121241.4) at position 544 nt, and the updated transcript contains an additional nucleotide at two sites (i.e., 1,303 nt and 1,342 nt). Gray regions indicate conserved nt sequence and black regions indicate nucleotide mismatches. (B) The amino acid sequences of *A. mellifera* probable cyclin-dependent serine/threonine-protein kinase (MF116383), *A. cerana* (XP_016906623.1), *A. dorsata* (XP_006616974.1), and *A. mellifera* (XP_001121241.2) were aligned using Geneious (Blosum62 parameters). The translated *A. mellifera* transcript described herein aligned the best with the *A. cerana* probable cyclin-dependent serine/threonine-protein kinase sequence with 1,234 identical amino acids (81.5% identity with gaps totaling 174 aa).

Together nucleotide and amino acid alignments indicate that the LOC725387 RNASeq consensus is most similar to *A. cerana* probable cyclin-dependent serine/threonine-protein kinase (XM_017051141.1), therefore, we refer to this gene as *A. mellifera* probable cyclin-dependent serine/threonine-protein kinase and deposited the sequence in GenBank Accession MF116383.

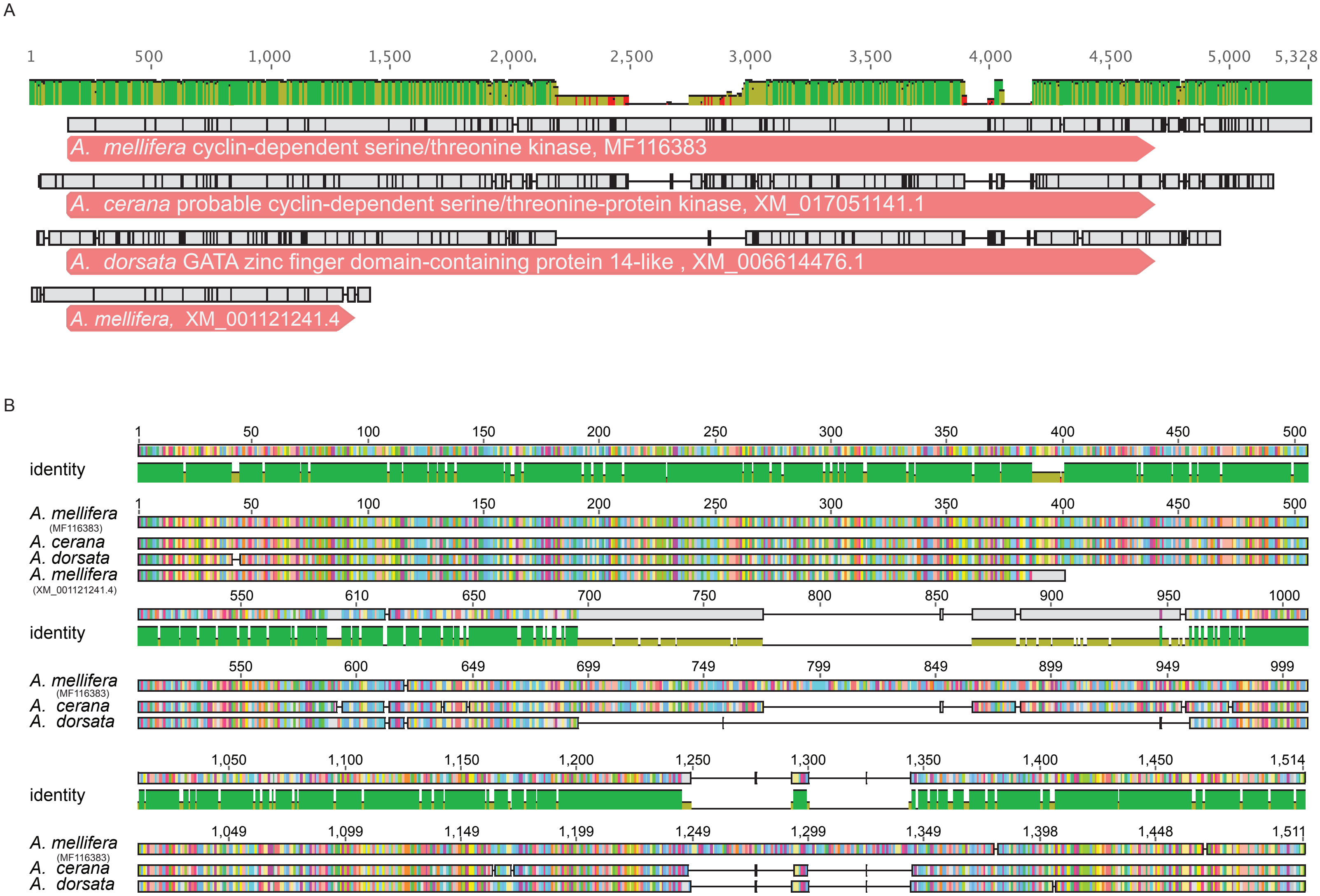

A
1
500
1,000
1,500
2,000
2,500
3,000
3,500
4,000
4,500
5,000
5,328
A. mellifera cyclin-dependent serine/threonine kinase, MF116383
A. cerana probable cyclin-dependent serine/threonine-protein kinase, XM_017051141.1
A. dorsata GATA zinc finger domain-containing protein 14-like , XM_006614476.1
A. mellifera, XM_001121241.4
B
identity
A. mellifera
(MF116383)
A. cerana
A. dorsata
A. mellifera
(XM_001121241.4)
identity
A. mellifera
(MF116383)
A. cerana
A. dorsata
identity
A. mellifera
(MF116383)
A. cerana
A. dorsata

S10 Fig. RNAi-mediated gene knockdown of dicer and cyclin-dependent kinase at 48 and 72 hpi resulted in increased virus abundance

To further investigate the role of *dicer* and probable *cyclin-dependent kinase* in honey bee antiviral defense, we utilized RNAi-mediated gene knock down to reduce their expression and qPCR to confirm reduced target gene expression and determine its impact on virus abundance (Fig 7). (A) In bees treated with dsRNA in the absence of virus at 48 and 72 hpi, *cyclin dependent kinase* expression was decreased in *kinase* specific dsRNA treated bees (spotted orange) by 46% (*p<0.05) and 50% (**p< 0.005) as compared to the ns-dsRNA controls (red horizontal lines). In virus and dsRNA-treated bees at 48 and 72 hpi, *cyclin dependent kinase* expression was decreased in *kinase* specific dsRNA treated bees (black diagonal lines) by 40% (*p<0.05) and 30% (*p< 0.05) as compared to the ns-dsRNA controls (checkered blue). (B) In bees treated with dsRNA in the absence of virus at 48 and 72 hpi, *dicer* expression was decreased in *dicer* specific dsRNA treated bees (vertical purple lines) by 56% (*p<0.05) and 31% (*p< 0.05) as compared to the ns-dsRNA controls (red horizontal lines). In virus-infected bees at 48 hpi, *dicer* expression was decreased in *dicer* specific dsRNA treated bees (spotted blue) by 32% (*p< 0.05), but it was not reduced at 72 hpi as compared to the ns-dsRNA controls. (C) The virus abundance of *kinase* and *dicer* specific dsRNA-treated bees had a trend of increased virus abundance compared to the ns-dsRNA controls, but this was not significant at 48 hpi. Virus abundance in *kinase* and dicer specific dsRNA-treated bees at 72 hpi was increased by 48% (*p< 0.05) and 44% (*p< 0.05) compared to the ns-dsRNA control. Relative gene transcript abundance was assessed using qPCR and ΔΔCT analysis, using *Am rpl8* as the house keeping gene; expression in each treatment group was compared to mock-infected controls. Percent relative virus abundance for each sample was determined via qPCR and ΔΔCT analysis using *Am rpl8* as the house keeping gene. Statistical differences between treatment (n=10) and virus-infected and ns-dsRNA bees were determined using one-sided Student's t-tests, *p< 0.05, **p< 0.005. The bars are standard error of the mean.

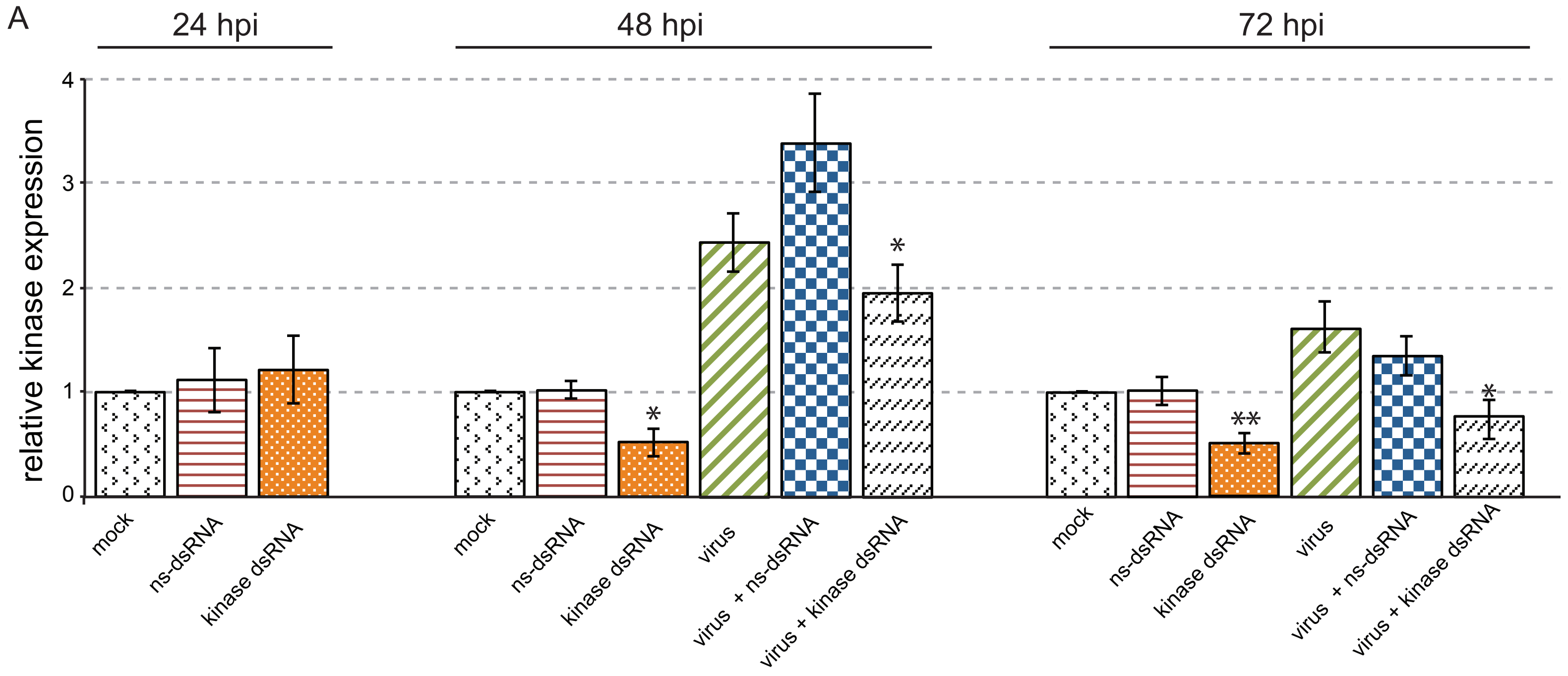

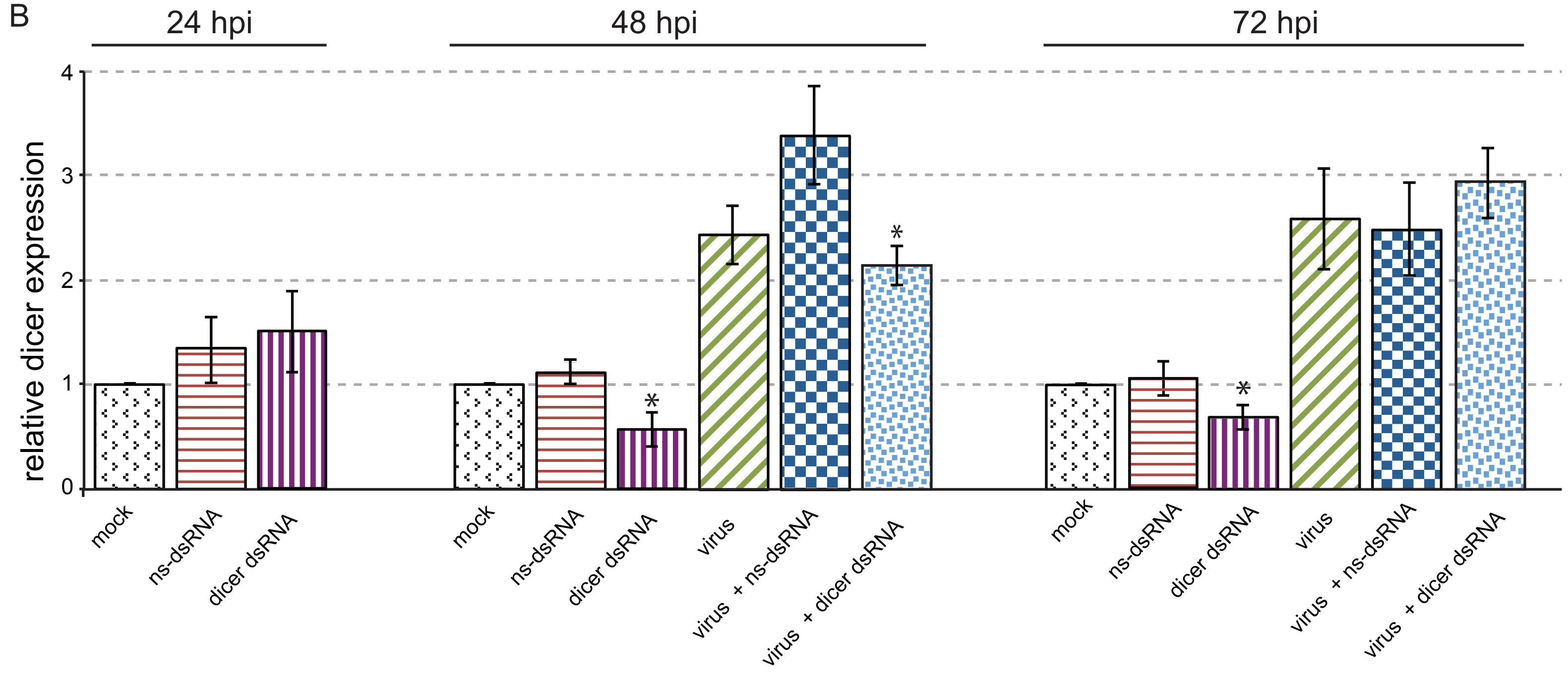

C

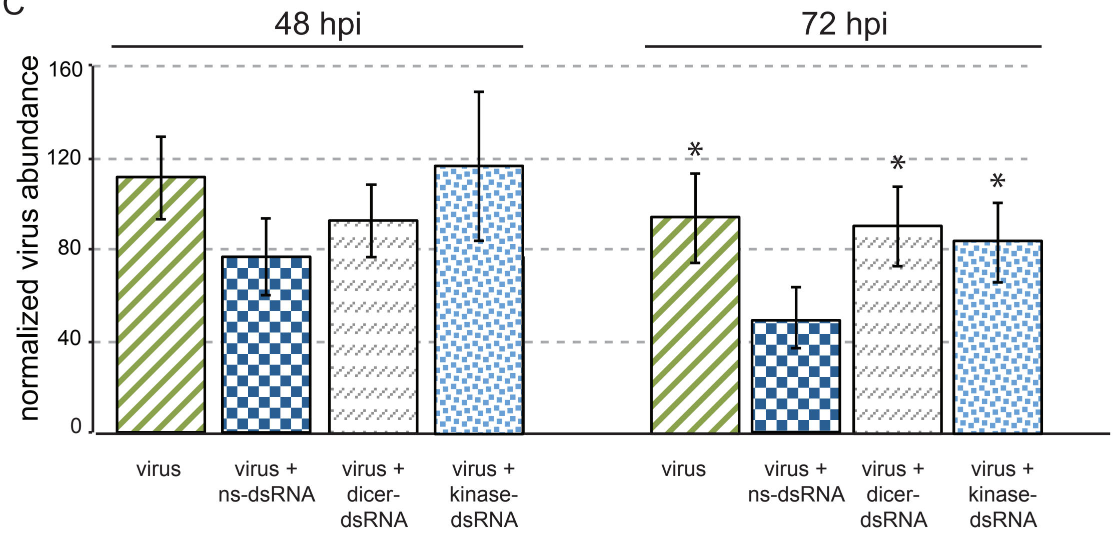
48 hpi
72 hpi
normalized virus abundance
160
120
80
40
0
*
*
*
virus
virus + ns-dsRNA
virus + dicer-dsRNA
virus + kinase-dsRNA
virus
virus + ns-dsRNA
virus + dicer-dsRNA
virus + kinase-dsRNA

Virus and dsRNA-triggered transcriptional responses reveal key components of honey bee antiviral defense

Laura M. Brutscher[1,2,3], Katie F. Daughenbaugh[1,3], and Michelle L. Flenniken[*,1,3]
[1]Department of Plant Sciences and Plant Pathology, [2]Department of Microbiology and Immunology, [3]Pollinator Health Center, Montana State University, Bozeman, MT, USA
*corresponding author: michelle.flenniken@montana.edu

Supplementary Methods

Honey bees

Frames of newly emerging bees were obtained from honey bee colonies maintained at Montana State University in Bozeman, MT, USA. Young (~ 24 hours post-emergence) female worker bees were utilized for experiments. The bees were housed in modified deli-containers at 32ºC and provided water and bee candy (i.e., powder sugar and corn syrup) for the duration of the experiment[1,2].

Sindbis virus (SINV-GFP) infection trials

Currently, there are no infectious honey bee virus clones, and though studies with semi-purified honey bee virus preparations have provided valuable information[3–5], we utilized a recombinant model virus, Sindbis virus expressing green fluorescent protein (SINV-GFP)[1,6]. There are several advantages to utilizing this virus including the ability to control the dose of virus inoculum, monitor the progression of virus infection using GFP, and the assurance that the honey bees were not previously infected with, nor exposed to, SINV-GFP. In addition, Sindbis virus does not encode a suppressor of RNAi (VSR)[7]. We and others have used SINV-GFP to investigate honey bee[1], fruit fly[6], and mosquito[8] antiviral defense mechanisms, thus facilitating comparison of immune responses in both natural mosquito hosts and non-native hosts (i.e., honey bee and fruit fly) that have not co-evolved with this virus. In brief, honey bees were immobilized via incubation at 4ºC for 20 minutes and injected in the thorax with 3,750 plaque forming units (PFUs) of SINV-GFP[1] diluted in 2 µl of 10 mM Tris buffer (pH 7.5) using a Harbo large capacity syringe equipped with disposable needles (Honey Bee Insemination Service; http://www.honeybeeinsemination.com/equipment2.html). The needles were prepared from borosilicate capillary tubes (0.8-1.10 x 100 mm) with a micropipette puller (Narishige Model PC-10, East Meadow, New York, USA). To investigate the role of dsRNA in honey bee antiviral defense, SINV-GFP was inoculated with multiple species and lengths of dsRNA (1 µg each), including virus-specific dsRNA (sp-dsRNA, 928 bp), nonspecific dsRNA corresponding to Drosophila C virus sequence (ns-dsRNA, 1,017 bp), and luciferase sequence (LUC dsRNA, 355 bp) (Supplementary Table S1). Bees were also co-injected with 1 µg high molecular weight polyinosinic-polycytidylic acid (poly(I:C)), InvivoGen, San Diego, California, USA), a synthetic mimic of dsRNA, or 1 µg nucleoside triphosphates (NTP), the positive and negative controls, respectively. After injection, bees typically recovered after 5 minutes at room temperature. Bees rarely died after injection (i.e., < 6%) and if so death was attributed to poor injection technique, as it was not associated with the substance injected (e.g., buffer, dsRNA, virus) and those bees were removed from the study. Mock-infection controls were also performed. Bees

were collected at 6, 48, or 72 hours post-infection (hpi). This time course was established to examine both early and late immune responses within a time frame that allowed for virus dissemination and infection, while maintaining optimal conditions for bees housed within the laboratory setting[1]. For each experimental treatment two additional biological repetitions that utilized bees from different colonies were evaluated at 48 and 72 hpi.

dsRNA preparation
dsRNA was generated by *in vitro* transcription with T7 RNA polymerase[6,9]. T7 promoter containing dsDNA PCR-products (1-10 µg) were amplified using the primers listed in Supplementary Table S1, with the following thermocycler program: pre-incubation of 95ºC (5 min), 35 cycles of 95ºC (30 s), 60ºC (30 s), and 72ºC (1 min) followed by a final incubation at 72ºC (5 min), and sequence verified using Sanger sequencing. The PCR products served as templates for T7 polymerase transcription (100 µl reactions: NTPs (each 7.5 mM final), RNase OUT (40 units) (Invitrogen, Carlsbad, CA, USA), buffer (400 mM HEPES pH 7.5, 120 mM MgCl2, 10 mM Spermidine, 200 mM DTT); reactions were carried out at 37ºC overnight (8-10 hours). DNA templates were removed by incubating with RQ1 DNAse (1 unit; Promega, Madison, WI, USA) for 15 minutes at 37ºC. ssRNA products were ethanol precipitated, suspended in 200 µl Rnase-free water, and annealed by heating the reaction to 100ºC for 5 minutes and then slowly cooling to room temperature. dsRNA products were purified by phenol:chloroform extraction followed by ethanol precipitation; dsRNA for injection was suspended in 10 mM Tris pH 7.5. dsRNA quality was assessed by agarose gel electrophoresis and spectrophotometer (NanoDrop 2000c, ThermoScientific, Waltham, MA, USA).The dsRNA quantity based on agarose gel band intensity was assessed using ImageJ[10].

dsRNA-mediated gene knockdown
The expression of two candidate antiviral genes: *dicer* (XM_016917734.1) and a novel transcript with 91% sequence identity with the *Apis cerana* probable cyclin-dependent serine/threonine-protein kinase DDB_G0292550 (XM_017051141.1), was reduced by RNAi-mediated gene knockdown (i.e., bees were injected with 1 µg of gene-specific dsRNA) (Supplementary Table S1). In order to assess the effects of gene knockdown on virus abundance, bees were infected with SINV-GFP (using methods as above) and co-injected with either gene-specific or nonspecific (DCV-specific) dsRNA (control).

Fluorescence Microscopy
The abdomens of individual honey bee bees were imaged using a Zeiss Stereoscope Stemi SV11 Apo, equipped with a Jenoptik Camera ProgResC14 Plus (Optical Systems GmBH P-07739 Jena). Fluorescence images were taken under fluorescent light with a GFP filter using standardized camera and exposure settings (i.e, 20x magnification, gain 60 and 150 ms exposure time). White light images were also taken 20x magnification (50 ms exposure).

Honey bee protein lysate preparation and analysis
Bees were dissected into head, thorax, and abdomen. The thoraxes and abdomens of bees collected at 72 hours post-infection were individually homogenized in 400 µl sterile H_2O with two sterile glass beads (5 mm) via bead beating for 1.5 minutes. The thorax

and abdomens of bees collected at 6 and 48 hours post-infection were individually homogenized in 400 µl sterile H_2O with one sterile 4.5 mm steel ball bearing using a Tissue Lyzer II (Qiagen, Hilden, Germany) for 1 minute. Lysates were clarified by spinning for 12 minutes at 12,000 x g.

For Western blot analysis, protein-containing honey bee lysates were combined with Laemmli buffer (95ºC for 3 min), electrophoresed on 12% acrylamide gels (Mini-PROTEAN TGX, BioRad, Hercules, CA), transferred to Immobilon-P PVDF membranes (EMD Millipore Corporation, Billerica, MA, USA), blocked with 5% milk in TBS buffer containing 0.1% Tween-20, incubated at 4ºC overnight with primary antibodies: either α-GFP (sc-8334; Santa Cruz Biotech, Santa Cruz, CA, USA) or α-β-actin (#4967L; Cell Signaling Technology, Danvers, MA, USA), washed, and then incubated with Horseradish peroxidase-conjugated anti-rabbit secondary antibody (ECL, GE Healthcare) for one hour at room temperature. The Western blots were developed with SuperSignal West Femto Maximum Sensitivity Substrate (Pierce, ThermoScientific, Waltham, MA, USA) and imaged with the Syngene G:Box F3 (software: G:Box Chemi-XR5). ImageJ[10] was used to quantify the sum of the pixels within β-actin and GFP regions in order to calculate and compare SINV-GFP levels in samples. The number of pixels within GFP regions were normalized based on the number of β-actin pixels as a loading control. Treatment groups in each Western blot set were assessed for statistical differences using Wilcoxon ranked-sum test.

Honey bee RNA isolation and purification
TRizol reagent (Invitrogen), 400 µl was added to 400 µl of individual bee thorax and abdomen homogenate, and RNA was isolated according to manufacturer's instructions. Prior to gene expression analysis by RNASeq or qPCR, RNA was further purified using Qiagen RNAeasy columns including on column DNase Treatment (Qiagen) to remove DNA from samples. RNA was quantified using a Thermo Scientific NanoDrop 2000 Spectrophotometer (Waltham, MA, USA).

Reverse transcription / cDNA synthesis
Reverse transcription reactions (25 µl) were performed using 500 ng of total RNA and random hexamer primers (500 ng) (IDT, Coralville, IA) incubated with Maloney murine leukemia virus (M-MLV) reverse transcriptase (Promega, Madison, WI) for 1 hour at 37 °C, according to the manufacturer's instructions.

Quantitative PCR (qPCR)
Quantitative PCR was utilized to examine the relative abundance of virus and honey bee host gene expression in each sample using previously described methods that are in accordance with published guidelines[11]. All qPCRs reactions were performed in triplicate using 2 µL of cDNA as template. Each 20 µl reaction was composed of cDNA template, 1X SYBR Green (Invitrogen, Cat.57563), 1X Choice Taq Master Mix (Denville Scientific Inc., Holliston, MA), 3 mM $MgCl_2$, and forward and reverse primers (600 nM each). A CFX Connect Real Time instrument (BioRad, Hercules, CA) was utilized for qPCR, the thermo-profile for virus (e.g., SINV-GFP and BQCV) and *Apis mellifera rpl8* analyses consisted of a single pre-incubation 95ºC (3 min), 40 cycles of 95ºC (5 s), and

60ºC (20 s); primers listed in Supplementary Table S1. Positive and negative controls, including the use of RNA templates from no RT enzyme cDNA reactions, were included for all qPCR analyses and exhibited the expected results.

To quantify the viral RNA (i.e., genome and transcript) abundance in each sample target SINV-GFP qPCR amplicons were cloned into the pGEM-T (Promega) vector, as described in Flenniken and Andino et al. 2013[1]. Plasmid standards, containing from 10^9 to 10^3 copies per reaction, were used as qPCR templates to assess primer efficiency and generate the SINV-specific standard curve used to quantify the viral RNA copy numbers within this range of detection[1]. The qPCR primers for RNAseq validation were designed using Primer3Plus and typically designed to have 60ºC annealing temperatures[12] (Supplementary Table S1). Melt point analysis and 2% agarose gel electrophoresis ensured qPCR specificity[13]. Primer efficiencies were evaluated using qPCR assays of cDNA and plasmid dilution series, and calculated by plotting $\log_{10}$ of the concentration versus the crossing point threshold (C(t)) values and using the primer efficiency equation, $(10^{(1/\mathrm{Slope})-1})$ x 100 (Supplementary Table S16).

The ΔΔC(t) method was used to calculate relative abundance of Sindbis-GFP in individual bees because it was most accurate since this method ensures that results are not skewed by inadvertent differences in RNA reverse-transcription efficiencies and starting cDNA template abundance[11,13,14]. Specifically, the ΔC(t) for each sample was calculated by subtracting the *Am rpl8* C(t) from the SINV-GFP C(t). The honey bee gene encoding ribosomal protein 8, *Am rpl8*, was selected as an appropriate housekeeping gene for qPCR, since it has been utilized in several other studies[15–18]. Analysis of the RNASeq data presented herein confirmed that *rpl8* expression was not significantly different in all sequenced libraries. The ΔΔC(t) was calculated by subtracting the average virus-infected ΔC(t) values from the ΔCt values for each treatment group. For host gene expression analyses and RNAseq validation, the percent gene expression for each gene of interest (GOI) was calculated using the following formula: 2^- ΔΔC(t) x100 = % gene expression, in which ΔC(t)=GOI C(t)- *rpl8* C(t), and ΔΔC(t)= sample ΔC(t) – mock-infected control ΔC(t).

To statistically examine the differences between the relative virus abundance of each treatment group for each time point, the SINV-GFP copy numbers and calculated relative abundance of each sample and was imported into R[19]. Based on previous work[1,20], we hypothesized that bees (n=10) co-injected with dsRNA or poly(I:C) would have decreased relative virus abundance as compared to the virus-only treated group. To examine relative virus abundance between treatment groups (e.g., virus-infected bees an dsRNA or poly(I:C) co-treated bees) that had equal variance and normal distribution we performed one-tailed students t-tests. Analysis of honey bee host gene expression revealed unequal variance between treatments groups and thus Welch's t-tests were used to identify statistical differences in host gene expression.

RNAseq Library Preparation and Sequencing

Bees were obtained from honey bee colonies which are subject to naturally occurring infections that may confound transcriptional results[21], so individual bee cDNA was

screened for pre-existing infections via PCR for several honey bee pathogens (Supplementary Table S3) using the following PCR thermocycler protocol: 95 °C (5 min); 35 cycles of 95 °C (30 s), 57 °C (30 s), and 72 °C (30 s), followed by final elongation at 72 °C for 4 minutes. If the sample was positive for a pathogen, the quantity was then assessed using qPCR. The RNA isolated from the abdomens of at least three representative bees with low (< 2,000 DWV and/or BQCV virus genome copies versus 7 x 10^4 - 7 x 10^6 SINV-GFP copies) to no pre-existing infections for each treatment group and time point were selected for transcriptome sequencing for a total of 47 individual bees (Supplementary Table S3). The abdomen was selected as the tissue of interest for RNASeq analysis of honey bee immune responses since virus infections would have naturally disseminated into this site post inoculation via intra-thoracic injection and it houses the fat body, which is an important tissue for immune function.

Prior to RNASeq library preparation, RNA from each sample was further purified using Qiagen RNeasy columns, including on-column DNase Treatment (Qiagen). The RNA quality was assessed using an Agilent 2200 Bioanalyzer (Santa Clara, CA, USA) and quantified with a Thermo Scientific NanoDrop 2000 Spectrophotometer (Waltham, MA, USA). The RNA was sent to Roy J. Carver Biotechnology Center at the University of Illinois at Urbana–Champaign for library preparation (Illumina TruSeq Stranded RNA Sample Prep kit). The libraries were prepared and pooled by experimental time point (15-17 samples per pool) in equimolar concentration and was quantitated using an Illumina Library quantification kit (Kapa). Each pool was sequenced on one lane for 101 cycles from each end of the fragments on a HiSeq2500 using a TruSeq SBS sequencing kit version 4. Sequencing yielded ~12 million reads per sample, which corresponding to at least 9.7 fold coverage using the standard equation that is used for genome / RNASeq coverage[22] (i.e., coverage = number reads (12 x 10^6 reads) x average read length (200bp) / length of genome (246.927 million bp for honey bee[23])) (Supplementary Table S2), which is in the range of coverage reported in other honey bee transcriptome studies[3,4,24]. Previous work has shown that sequencing as few as 1 million reads can provide significant and valuable information of differential expression patterns in honey bees[24]. Sequence data was deposited into the NCBI Sequence Read Archive under accession number SRP101337 and is also linked with NCBI BioProject #PRJNA377749.

Specific code used for RNAseq analysis is listed below. Briefly, the programs FastQC and fastx-toolkit were used to remove low quality reads (<Q30). Illumina adapters were trimmed with Trimmomatic[25], and reads were aligned to the *A. mellifera* genome assembly 4.5 (amel4.5.fa) from NCBI with Tophat v2.0.14[26]; on average, ~77% of reads from each sample mapped to the *Apis mellifera* genome (Supplementary Table S2). To determine the normalized number of fragments per Fragments Per Kilobase of transcript per Million mapped reads (FPKM) and compare expression between treatments, mapped reads were analyzed with CuffDiff[27,28] using default parameters: FPKM normalization (i.e., library size factor is set to 1 - no scaling applied to FPKM values or fragment counts), pooled cross-replicate dispersion estimation method (i.e., each replicated condition is used to build a model, then these models are averaged to provide a single global model for all conditions in the experiment), and a Benjamini-

Hochberg correction q-value cutoff at ≤0.05,. The transcriptome profiles of replicates from each treatment (i.e., mock-injected controls, virus, dsRNA, virus and specific dsRNA, virus and nonspecific dsRNA) and time point (6, 48, or 72 hpi) were analyzed individually. CuffDiff results were interpreted graphically using CummeRbund[29] in R. Genes were considered significantly differentially expressed if they had a Benjamini-Hochberg corrected q-value ≤ 0.05. To assess shared differentially expressed genes (DEGs) between comparisons and create Venn diagrams of shared DEGs, Cuffdiff output was processed with the program Vennt[30]. We were primarily concerned with genes that were differentially expressed between mock-injected controls and treatments (i.e., virus, dsRNA, virus and specific dsRNA, virus and nonspecific dsRNA) at each time point (6, 48, or 72 hpi), and so the DEGs and their respective q-values are listed in Supplementary Table S5. On average, differentially expressed genes (q-value<0.05, total of 2,830 genes) from all pair-wise comparisons of interest had a count of 37,924 reads across all samples, with a median of 8,757 reads and range of 92-3,327,734 read counts per gene.

To further investigate the function of the DEGs, representative protein sequences (the longest sequence if there were splice variants) of every known honey bee gene were queried against the *D. melanogaster* protein database via reciprocal BLAST+[31] to identify fruit fly orthologs and homologs of the honey bee genes there is a greater amount of gene ontology information for *D. melanogaster* genes compared to *Apis mellifera* genes. The honey bee genome encodes approximately 15,000 genes of which 13,592 genes are mapped and provided in the Amel4.5 genome annotation[23]. We annotated 8,944 genes (~66%) as homologs (of which 7,006 were reciprocal best hits or orthologs) to genes encoded by the fruit fly *D. melanogaster* genome, which encodes ~13,600 genes [23,32]. Biological processes (BP) functional enrichment analysis was performed with DAVID[33].

Comparative analysis of DEGs in virus-infected bees

To identify the shared and unique DEGs in virus-infected bees, we compared our dataset to other studies that examined gene expression in virus-infected bees. We compared the genes that were differentially expressed in SINV-GFP infected bees 72 hpi to DEGs in symptomatic IAPV-fed bees[3], SBV and DWV-infected bees[4], adult honey bees naturally infected with IAPV[34], and a common DEG list that was compiled from 19 gene expression data sets including *Varroa destructor*-parasitized and virus-infected bees[35]. We used NCBI gene ID as a common identifier because DEG lists were generated using different technologies (i.e., microarray and high throughput sequencing) and versions of the *Apis mellifera* genome and transcriptome. The DEGs in SBV and DWV-infected bees described by Ryabov et al.,[4] were correlated to our reference gene list using the BeeBase Bee IDs, and resulted in the common identification of 1,315 of the 1,638 DEGs. Similarly, we assigned common gene identifiers for 662 of the 753 differentially regulated transcripts in the symptomatic IAPV-fed bees [3]. The DEGs identified in naturally IAPV infected adult honey bees using microarray analysis [34] was kindly provided by Dr. Judy Chen. This list consisted of microarray probe IDs, therefore, in order to compare the microarray data to our data set, the microarray oligonucleotide probe sequences were aligned to *Apis mellifera* refseq

RNAs using BLAST (megablast, plus / plus strands) and the corresponding NCBI Entrez Gene IDs were identified. We identified 4,402 NCBI Entrez Gene IDs corresponding to the 5,637 probes for specific transcripts and transcript variants. Of the 4,402 probes for which we identified 4,307 Gene IDs, 3,109 of them matched distinct genes that were also in our gene list. The NCBI Entrez Gene IDs of each DEG list (Supplementary Table S14) were then compared via Venn diagram analysis[36](Supplementary Table S15). Pairwise comparisons between studies identified shared DEGs and the statistical significance of gene overlap was assessed using hypergeometric tests[37] (Supplementary Table S15).

Identification of previously unrecognized transcript

RNASeq analysis determined that reads aligning to LOC725387 were more abundant in virus-infected bees. To identify the gene or genes encoded by these differentially expressed reads, the consensus nucleotide sequence was used to query the NCBI Nucleotide collection (nr/nt) and *A. mellifera* databases using blastn[38], Sanger sequencing was performed to verify transcript sequence and length, and the results were evaluated using Geneious[39]. Together, these analyses revealed that we identified a previously unrecognized transcript, *A. mellifera* probable cyclin-dependent serine/threonine-protein kinase (MF116383, 5,158 nt), which is longer the originally annotated *A. mellifera* probable serine/threonine-protein kinase clkA (LOC725387, XM_001121241.4, 1,403 nt).

In brief, we utilized LOC725387 RNASeq consensus sequence to query the NCBI Nucleotide nr/nt data base and identified an *A. cerana* transcript annotated as a probable cyclin-dependent serine/threonine-protein kinase DDB_G0292550 (LOC107994302, XM_017051141.1) as the top blastn result, which contained 95% of the submitted sequence and shared 91% identity (E-value = 0, 95% query coverage, 91% identity, 1-6% gaps); additional top blastn hits included *A. dorsata* GATA zinc finger domain-containing protein 14-like. When the LOC725387 RNASeq consensus sequence was used to query the *A. mellifera* database, the top blastn result only covered 24% of the query sequence (i.e., *A. mellifera* probable serine/threonine-protein kinase clkA, XM_001121241.4; E-value = 0, 24% query coverage, 99% identity, 0% gaps). To further characterize the LOC725387 transcript, we Sanger sequenced 5,027 nts (~2–3x coverage) and obtained the most 5'end of this transcript from RNASeq data (131 bp, >2,000x coverage) (Supplementary Table S1 and Fig. S9). Together nucleotide and amino acid alignments indicate the RNAseq reads aligning to LOC725387 are most similar to a computationally predicted *A. cerana* cyclin-dependent serine/threonine-protein kinase DDB_G0292550 (Supplementary Figure S9), therefore, we refer to the gene identified herein as *A. mellifera probable cyclin-dependent serine/threonine-protein kinase* (Supplementary Fig. S9) and submitted the sequence of this transcript to NCBI (MF116383).

Supplementary RNAseq Analysis Code

Sequencing and raw data information

- 100 nt paired-end reads sequenced on the Illumina HiSeq2500
- Average cDNA fragment size: 230nt (range from 80nt - 700nt)
- The stranded RNAseq libraries were prepared with Illumina's 'TruSeq Stranded RNA Sample Prep kit'.
- The libraries were pooled in equimolar concentration and the pool was quantitated by qPCR and sequenced on one lane for 101 cycles from each end of the fragments

Programs in order of use

1. **FastQC** Version 0.11.3

   Example code

   ```
   fastqc SAMPLE _ATCACG_L005_R1_001.fastq
   ```

2. **Trimmomatic** Version 0.32

   Example code

   ```
   trimmomatic PE -phred33 -threads 4 –trimlog SAMPLE_trimlog SAMPLE
   _ATCACG_L005_R1_001.fastq
   SAMPLE_ATCACG_L005_R2_001.fastq  SAMPLE_F_paired.fastq
   SAMPLE_F_unpaired.fastq SAMPLE_48hr_R_paired.fastq SAMPLE
   _48hr_R_unpaired.fastq ILLUMINACLIP:TruSeq3-PE-2.fa:2:10:10:6
   LEADING:3 TRAILING:3 SLIDINGWINDOW:4:15 MINLEN:36
   TOPHRED33
   ```

3. **FastQC** Version 0.11.3
   a. Perform on resultant trimmomatic fastq and assess “Adapter Content” statistic for remnant adaptors in file.

4. **Bowtie2 build** (version 2.2.5.0)

   Example code

   ```
   bowtie2-build ./GCF_000002195.4_Amel_4.5.fna ./
   GCF_000002195.4_Amel_4.5.scaffolds

   bowtie2-inspect --summary ./ GCF_000002195.4_Amel_4.5.scaffolds >
   GCF_000002195.4_Amel_4.5.scaffolds.summary
   ```

5. **Tophat2** Version 2.0.14.OSXx86_64

Example code

```
tophat2 -p 8 --mate-inner-dist 200 --library-type fr-firststrand --max-multihits 1 --mate-std-dev 100 -o ./tophat2_out_SAMPLE GCF_000002195.4_Amel_4.5.scaffolds SAMPLE_F_paired.fastq SAMPLE_R_paired.fastq, SAMPLE_R_unpaired.fastq, SAMPLE_F_unpaired.fastq;
```

6. **Genome Browsers- Integrated Genome Viewer (IGV)**
    a. Allows visualization of reads aligning to genome/transcriptome using alignment files, “accepted_hits”, generated from Tophat2.
    b. Use it to confirm:
        i. library type choice looks correct.
        ii. Lack of widespread exact read duplicates (i.e. probably library PCR depth issues).
        iii. 5' / 3' density of reads, possible library or RNA quality biases.

7. **Samtools**
    a. Can be used to convert bam to sam or sam to bam if desired.
    b. IGV requires the file to be in sam format.

Example code

```
samtools view sample_accepted_hits.bam > 3_a_accepted_hits.sam
```

8. **Cuffdiff** Version 2.1.1.OSX_x86_64

Example code

```
cuffdiff -o cuffdiff_ALL_timepoints --num-threads 5 --labels
buffer_6hr,virus_6hr,buffer_dsRNA_6hr,virus_specific_dsRNA_6hr,virus_
nonspecific_dsRNA_6hr,buffer_48hr,virus_48hr,buffer_dsRNA_48hr,viru
s_specific_dsRNA_48hr,virus_nonspecific_dsRNA_48hr,buffer_72hr,viru
s_72hr,buffer_dsRNA_72hr,virus_specific_dsRNA_72hr,virus_nonspecifi
c_dsRNA_72hr --library-type fr-firststrand
GCF_000002195.4_Amel_4.5_genomic.gff
2a_6hr.bam,2c_6hr.bam,2e_6hr.bam
3b_6hr.bam,3f_6hr.bam,3i_6hr.bam
4a_6hr.bam,4b_6hr.bam,4e_6hr.bam
5b_6hr.bam,5d_6hr.bam,5e_6hr.bam
7d_6hr.bam,7f_6hr.bam,7g_6hr.bam
2b_48hr.bam,2d_48hr.bam,2g_48hr.bam
3a_48hr.bam,3f_48hr.bam,3h_48hr.bam
4a_48hr.bam,4b_48hr.bam,4c_48hr.bam
5b_48hr.bam,5c_48hr.bam,5l_48hr.bam
7g_48hr.bam,7h_48hr.bam,7i_48hr.bam
2a_72hr.bam,2b_72hr.bam,2d_72hr.bam 3d_72hr.bam, 3e_72hr.bam
3h_72hr.bam,3i_72hr.bam,3j_72hr.bam
4f_72hr.bam,4h_72hr.bam,4i_72hr.bam
5b_72hr.bam,5b_72hr_RII.bam,5c_72hr.bam
7b_72hr.bam,7e_72hr.bam,7i_72hr.bam
```

9. **Vennt**
   a. Download python script "venn.py" from http://drpowell.github.io/vennt/
   b. Works with Cuffdiff output and generates list of DEGs based on manually set q values, for which it was set to $\leq$0.05.

Example code

```
python vennt.py --cuffdiff gene_exp.diff > RNAseq-vennt.html
```

References

1. Flenniken, M. L. & Andino, R. Non-specific dsRNA-mediated antiviral response in the honey bee. *PLoS One* **8,** e77263 (2013).
2. Evans, J. D., Chen, Y. P., Prisco, G., Pettis, J. & Williams, V. Bee cups : single-use cages for honey bee experiments. **48,** 300–302 (2009).
3. Galbraith, D. a., Yang, X., Niño, E. L., Yi, S. & Grozinger, C. Parallel Epigenomic and Transcriptomic Responses to Viral Infection in Honey Bees (Apis mellifera). *PLOS Pathog.* **11,** e1004713 (2015).
4. Ryabov, E. V, Fannon, J. M., Moore, J. D., Wood, G. R. & Evans, D. J. The Iflaviruses Sacbrood virus and Deformed wing virus evoke different transcriptional responses in the honeybee which may facilitate their horizontal or vertical transmission. *PeerJ* **4,** e1591 (2016).
5. Brutscher, L. M., Daughenbaugh, K. F. & Flenniken, M. L. Antiviral defense mechanisms in honey bees. *Curr. Opin. Insect Sci.* **2,** 1–12 (2015).
6. Saleh, M., Tassetto, M., Rij, R. Van & Goic, B. Antiviral immunity in Drosophila requires systemic RNA interference spread. *Nature* **458,** 346–350 (2009).
7. Nayak, A. *et al.* Cricket paralysis virus antagonizes Argonaute 2 to modulate antiviral defense in Drosophila. *Nat. Struct. Mol. Biol.* **17,** 547–554 (2010).
8. Venticinque, L. & Meruelo, D. Sindbis viral vector induced apoptosis requires translational inhibition and signaling through Mcl-1 and Bak. *Mol. Cancer* **9,** 37 (2010).
9. Amdam, G. V, Simões, Z. L. P., Guidugli, K. R., Norberg, K. & Omholt, S. W. Disruption of vitellogenin gene function in adult honeybees by intra- abdominal injection of double-stranded RNA. *BMC Biotechnol.* **8,** 1–8 (2003).
10. Schneider, C. a, Rasband, W. S. & Eliceiri, K. W. NIH Image to ImageJ: 25 years of image analysis. *Nat. Methods* **9,** 671–675 (2012).
11. Huggett, J. F. *et al.* The digital MIQE guidelines: Minimum information for publication of quantitative digital PCR experiments. *Clin. Chem.* **59,** 892–902 (2013).
12. Untergasser, A. *et al.* Primer3-new capabilities and interfaces. *Nucleic Acids Res.* **40,** 1–12 (2012).
13. Ginzinger, D. G. Gene quantification using real-time quantitative PCR: an emerging technology hits the mainstream. *Exp. Hematol.* **30,** 503–12 (2002).
14. Scientific, T. *Real-time PCR*. *Scientific, ThermoFisher* (2016).
15. Evans, J. D. Beepath: an ordered quantitative-PCR array for exploring honey bee immunity and disease. *J. Invertebr. Pathol.* **93,** 135–9 (2006).
16. Hou, C. S., Li, B. B., Deng, S. & Diao, Q. Y. Effects of Varroa destructor on temperature and humidity conditions and expression of energy metabolism genes in infested honeybee colonies. *Genet. Mol. Res.* **15,** (2016).
17. Hou, C., Rivkin, H., Slabezki, Y. & Chejanovsky, N. Dynamics of the presence of israeli acute paralysis virus in honey bee colonies with colony collapse disorder. *Viruses* **6,** 2012–27 (2014).
18. De Smet, L. *et al.* BeeDoctor, a versatile MLPA-based diagnostic tool for screening bee viruses. *PLoS One* **7,** e47953 (2012).
19. Team, Rs. RStudio: Integrated Development for R. (2015).
20. Niu, J., Meeus, I. & Smagghe, G. Differential expression pattern of Vago in bumblebee (Bombus terrestris), induced by virulent and avirulent virus infections. *Sci. Rep.* **6,** 34200 (2016).
21. McMenamin, A. J. & Genersch, E. Honey bee colony losses and associated viruses. *Curr. Opin. Insect Sci.* **8,** 121–129 (2015).

22. Lander, E. S. & Waterman, M. S. Genomic mapping by fingerprinting random clones: A mathematical analysis. *Genomics* **2,** 231–239 (1988).

23. Elsik, C. G. *et al.* Finding the missing honey bee genes: lessons learned from a genome upgrade. *BMC Genomics* **15,** 86 (2014).

24. Atallah, J., Plachetzki, D. C., Jasper, W. C. & Johnson, B. R. The utility of shallow RNA-Seq for documenting differential gene expression in genes with high and low levels of expression. *PLoS One* **8,** 1–11 (2013).

25. Trimmomatic. a flexible trimmer for Illumina sequence data. *Bioinformatics* **30,** 2114 (2014).

26. Kim, D. *et al.* TopHat2: accurate alignment of transcriptomes in the presence of insertions, deletions and gene fusions. *Genome Biol.* **14,** R36 (2013).

27. Trapnell, C. *et al.* Differential gene and transcript expression analysis of RNA-seq experiments with TopHat and Cufflinks. *Nat. Protoc.* **7,** 562–78 (2012).

28. Trapnell, C. *et al.* Differential analysis of gene regulation at transcript resolution with RNA-seq. *Nat. Biotechnol.* **31,** 46–53 (2013).

29. Goff, L. A., Trapnell, C. & Kelley, D. CummeRbund: visualization and exploration of Cufflinks high-throughput sequencing data. *R Packag. Version 2.2* (2012).

30. Powell, D. R. Vennt : Dynamic Venn tool for differential gene expression. (2014). Available at: http://drpowell.github.io/vennt/. (Accessed: 1st August 2015)

31. Camacho, C. *et al.* BLAST plus: architecture and applications. *BMC Bioinformatics* **10,** 1 (2009).

32. Weinstock, G. M. *et al.* Insights into social insects from the genome of the honeybee Apis mellifera. *Nature* **443,** 931–949 (2006).

33. Dennis Jr, G. *et al.* DAVID: Database for Annotation, Visualization, and Integrated Discovery. *Genome Biol.* **4,** P3 (2003).

34. Chen, Y. P. *et al.* Israeli acute paralysis virus: epidemiology, pathogenesis and implications for honey bee health. *PLoS Pathog.* **10,** e1004261 (2014).

35. Doublet, V. *et al.* Unity in defence: honeybee workers exhibit conserved molecular responses to diverse pathogens. *BMC Genomics* **18,** 207 (2017).

36. Venn. Available at: http://bioinformatics.psb.ugent.be/webtools/Venn/). (Accessed: 3rd January 2017)

37. Alaux, C. *et al.* Honey bee aggression supports a link between gene regulation and behavioral evolution. *Proc. Natl. Acad. Sci.* **106,** 15400–15405 (2009).

38. Altschul, S. F., Gish, W., Miller, W., Myers, E. W. & Lipman, D. J. Basic local alignment search tool. *J. Mol. Biol.* **215,** 403–10 (1990).

39. Kearse, M. *et al.* Geneious Basic: An integrated and extendable desktop software platform for the organization and analysis of sequence data. *Bioinformatics* **28,** 1647–1649 (2012).
